# Tailoring Oxide/MAX Phase Nanocomposites via Low‐Temperature Oxidation for Lithium‐Ion Battery Anodes: Peeking Behind the Electrochemical Mechanism via In Situ Investigations

**DOI:** 10.1002/advs.202512947

**Published:** 2025-09-24

**Authors:** Irene Ostroman, Nicholas Vallana, Antonio Gentile, Stefano Marchionna, Omar Perego, Chiara Ferrara, Andrew Fitch, Martina Fracchia, Nicolò Pianta, Andrea Giacomo Marrani, Taewon Kim, Changhyun Park, Chanhee Lee, Hyun‐Wook Lee, Lorenzo Stievano, Riccardo Ruffo

**Affiliations:** ^1^ Department of Materials Science Università degli Studi di Milano‐Bicocca Via Cozzi 55 Milano 20125 Italy; ^2^ Ricerca sul Sistema Energetico ‐ RSE S.p.A. Via R. Rubattino 54 Milano 20134 Italy; ^3^ European Synchrotron Radiation Facility 71 Avenue des Martyrs Grenoble 38000 France; ^4^ Department of Chemistry Università di Pavia Via T. Taramelli 12 Pavia 27100 Italy; ^5^ National Reference Center for Electrochemical Energy Storage (GISEL) Via G. Giusti 9 Firenze 50121 Italy; ^6^ INSTM Consorzio Interuniversitario per la Scienza e Tecnologia dei Materiali Via G. Giusti 9 Firenze 50121 Italy; ^7^ Department of Chemistry Università di Roma “La Sapienza” p.le A. Moro 5 Roma I‐00185 Italy; ^8^ Department of Materials University of Oxford Oxford OX1 3PH UK; ^9^ School of Energy and Chemical Engineering Ulsan National Institute of Science and Technology 50 UNIST‐gil Ulsan 44919 Republic of Korea; ^10^ ICGM CNRS, ENSCM Univ. Montpellier Montpellier 34293 France; ^11^ Réseau sur le Stockage Electrochimique de l'Energie (RS2E) CNRS Amiens 80039 France

**Keywords:** anodes, lithium ion batteries, MAX phases, nanostructured Ti Sn oxides, negative electrodes

## Abstract

This study explores the potential of MAX phase/oxide nanocomposites as negative electrodes for lithium‐ion batteries. The main objective is to enhance the stability and performance of tin oxide‐based electrodes by reducing volume changes upon cycling. The approach involves the synthesis of a Sn‐containing MAX phase (Ti_3_Al_0.3_Sn_0.7_C_2_) followed by oxidation at different temperatures (600, 700, and 850 °C). Comprehensive characterization reveals that partial oxidation produces nanocomposites containing titanium and tin oxide nanoparticles with different compositions depending on the annealing temperature. The residual presence of the MAX phase contributes to the stability of the electrode, buffering volume changes during cycling. The sample oxidized at 700 °C exhibits the best trade‐off between specific capacity (350 mAh g^−1^ at 50 mA g^−1^) and reversibility (99.2% Coulombic efficiency), and it delivers a reversible specific capacity of 133 mAh g^−1^ at 2000 mA g^−1^ which is superior to the high‐rate performance typically reported for graphite. In situ studies provide insights into the mechanism of (de)lithiation, confirming the reduction of Sn(IV) to metallic Sn and the subsequent formation of Li‐Sn alloys, while the residual MAX remains electrochemically inactive, preserving structural integrity and transport properties.

## Graphical abstract

Controlled oxidation of Ti_3_Al_0.3_Sn_0.7_C_2_ produces SnO_2_/TiO_2_–MAX nanocomposites with superior electrochemical performance. The SnHigh_Ox700 sample shows optimal stability, capacity (300 mAh g^−1^), and reversibility (99.2%). Its unique morphology ensures efficient charge transport and stress accommodation. In situ analyses reveal reversible SnO_2_ conversion/alloying, while MAX cores stabilize cycling, enabling strong full‐cell performance versus graphite.

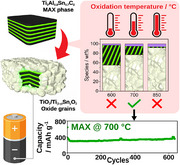

## 1 Introduction

Lithium‐ion batteries (LIBs) are the most widely used energy storage devices due to their high energy density, reliability, and performance. Graphite remains the standard negative electrode, with a theoretical capacity of 372 mAh g^−1^.^[^ ^1^, ^2^ ^]^ However, its low lithiation potential (≈0.1 V vs Li⁺/Li) limits fast‐charging performance and increases safety risks, as high currents can induce non‐uniform lithiation and lithium metal plating.^[^ ^3^, ^4^, ^5^, ^6^, ^7^, ^8^ ^]^ Therefore, there is a growing need to identify safer materials as possible negative electrodes.^[^ ^9^ ^]^ This effect, exacerbated at higher rates, underscores the need for safer, high‐performance alternatives. Alloy‐based anodes, such as lithium‐silicon and lithium‐tin, offer higher capacities (3579 and 993 mAh g^−1^, respectively) and operate at higher potentials, reducing plating risks.^[^ ^4^, ^10^, ^11^ ^]^ Yet, their practical application is challenged by large volume changes during cycling, causing mechanical stress, particle fracture, and unstable solid electrolyte interphases (SEIs), which compromise cycle life.^[^ ^12^, ^13^, ^14^, ^15^, ^16^ ^]^ One possible way to exploit the alloying reactions and reduce mechanical instability is to use oxides (SiO_x_, SnO_x_) instead of bare metals.^[^ ^17^, ^18^ ^]^ During the initial reduction reaction, the oxide reacts with lithium ions in a conversion mechanism to produce the corresponding metal and lithium oxide Li_2_O:

(1) $$MO_{x}+2x\;Li^{+}+2x\;e^{-}\to M+x\;Li_{2}O$$

This reduction is then followed by the alloying reaction:

(2) $$M+y\;Li^{+}+y\;e^{-}⇄Li_{y}M$$

This strategy produces an intimately mixed composite of metal particles embedded in a Li_2_O matrix, which can help buffer the volume changes, but has also the disadvantage of a largely irreversible first‐cycle capacity loss due to the conversion reaction.^[^ ^19^ ^]^ The conversion reaction itself can be at least partially reversible, depending on the nanometric size of the particles, thus increasing the reversible capacity of the electrode.^[^ ^20^, ^21^, ^22^ ^]^ Beyond mechanical stabilization, oxide‐based strategies also enhance SEI stability. For example, oxycarbides form near‐zero‐strain interphases that suppress lithium silicate formation and accommodate crystalline phases without inducing structural failure.^[^ ^23^ ^]^ Likewise, nitrogen treatment enhances surface lithiophilicity, promoting the formation of robust and uniform SEI layers.^[^ ^24^ ^]^

In the pursuit of enhancing the durability of these promising materials, researchers have explored and developed two primary strategies. The former involves leveraging nanostructures, such as nanowires and nanorods:^[^ ^25^, ^26^, ^27^, ^28^, ^29^ ^]^ smaller particle sizes facilitate the stress release, capitalizing on the available free porosity around the nanoparticles, consequently yielding improved rate performance.^[^ ^30^ ^]^ Such an approach entails the creation of composite electrodes with intimate contacts between the conversion oxide and conducting agents (graphene, carbon nanotubes, MXenes), which can also buffer the volume changes.^[^ ^13^, ^27^, ^31^, ^32^, ^33^, ^34^, ^35^, ^36^, ^37^ ^]^ A less explored strategy involves the use of solid solutions composed of various oxides. In this case, conversion oxide, such as SnO_2_, is combined with an intercalation oxide, i.e., TiO_2_, that can serve as a robust framework. And mitigate volume change observed during cycling, thereby contributing to overall electrode durability.^[^ ^38^, ^39^, ^40^, ^41^ ^]^

In this contest, we have recently introduced an innovative approach to the preparation of nanocomposites for lithium batteries, namely the thermal oxidation of MAX phases to obtain nanostructured mixtures of oxide supported on a conductive scaffold.^[^ ^42^, ^43^, ^44^ ^]^ MAX phases are a class of 3D materials, with stoichiometric formula M_n+1_AX_n_ (where M is a transition metal, A is a metallic or metalloid element, X is boron, nitrogen, or carbon, and n equals 1, 2, 3, or 4), characterized by a hexagonal layered crystal structure (space group P6_3_/mmc), consisting of several layers of M_6_X octahedra alternated with layers of pure A‐elements along the c parameter.^[^ ^45^, ^46^ ^]^ MAX phases present strong covalent M‐X bonds on the layers, with much weaker bonds between these layers and the A‐element; therefore, they exhibit a structural feature similar to graphite. Thanks to this, they exhibit properties of both metals and ceramic materials, i.e., high electric and thermal conductivity, as well as chemical stability like ceramics.^[^ ^47^, ^48^ ^]^ Among different applications, the most common MAX phase Ti_3_AlC_2_ was also used as an electrode material versus lithium, but it showed negligible energy storage performance.^[^ ^47^, ^49^ ^]^

The concept of using a specifically designed MAX phase to obtain a nanostructured composite electrode based on oxide active materials such as TiO_2_ and SnO_2_ revolves around the direct derivation of the two oxides from the MAX phase. This is obtained by finely tuning the pristine MAX composition followed by controlled oxidation, thereby combining the favorable electrochemical performance of SnO_2_ with the robust mechanical properties of TiO_2_ and the unique structural and conductive features of the MAX phase. This synergistic combination fortifies the entire system, enabling it to withstand volume changes effectively. Moreover, our objective encompasses the in situ formation of oxides directly from the MAX phase, enabling their synthesis as nanostructured particles embedded within a 3D structure, thereby minimizing the likelihood of cracking.

In this framework, it has been recently shown that controlled oxidation of a Sn‐containing MAX phase (Ti_3_Al_1‐x_Sn_x_C_2_ with nominal composition x = 0.4 or 0.7) at 600 °C in air yields a composite material capable of very good electrochemical performance as a negative electrode in LIBs.^[^ ^43^ ^]^ Yet, despite these promising results, the fundamental oxidation mechanism and its direct impact on electrochemical behavior remain largely unexplored. Addressing this knowledge gap is essential, as a detailed understanding of the reaction pathway is the key to unlocking the full potential of this class of materials. In the present work, we clearly establish this novelty by providing, for the first time, a comprehensive investigation of the oxidation process of the most promising Sn‐rich MAX composition. The oxidation reaction is systematically studied as a function of temperature, combining in situ analyses (X‐ray Diffraction, XRD) with advanced characterization of the resulting oxides at three different temperatures (600, 700, and 850 °C) through X‐ray Photoelectron Spectroscopy (XPS), X‐ray Absorption Spectroscopy (XAS), Scanning and Transmission Electron Microscopy (SEM, TEM), and Mössbauer spectroscopy. Among these, the sample obtained at 700 °C demonstrates an outstanding compromise between specific capacity (330 mAh g^−1^ @ 100 mA g^−1^) and reversibility (99.2% charge efficiency) and is therefore investigated in greater depth. The electrochemical performance of this optimized material (capacity, efficiency, rate capability, and cycling stability) is thoroughly evaluated in both half‐cell (vs Li metal) and full‐cell configurations (vs commercial Ni‐rich LiNi_0_._8_Mn_0_._1_Co_0_._1_O_2_, NMC). Finally, the (de)lithiation mechanism is elucidated using a wide suite of complementary in situ (XAS, XRD, TEM) and ex situ (XPS, Mössbauer) techniques, providing unprecedented insights into the role and synergy of the different components within the electrode.

In the following, the samples will be labeled SnHigh. This notation is also used for the thermally treated samples, where the name of the compound is followed by “_Ox” and oxidation temperature, e.g., SnHigh_Ox700.

## 2 Results and Discussion

### 2.1 Study of the MAX Oxidation

**Figure 1:**
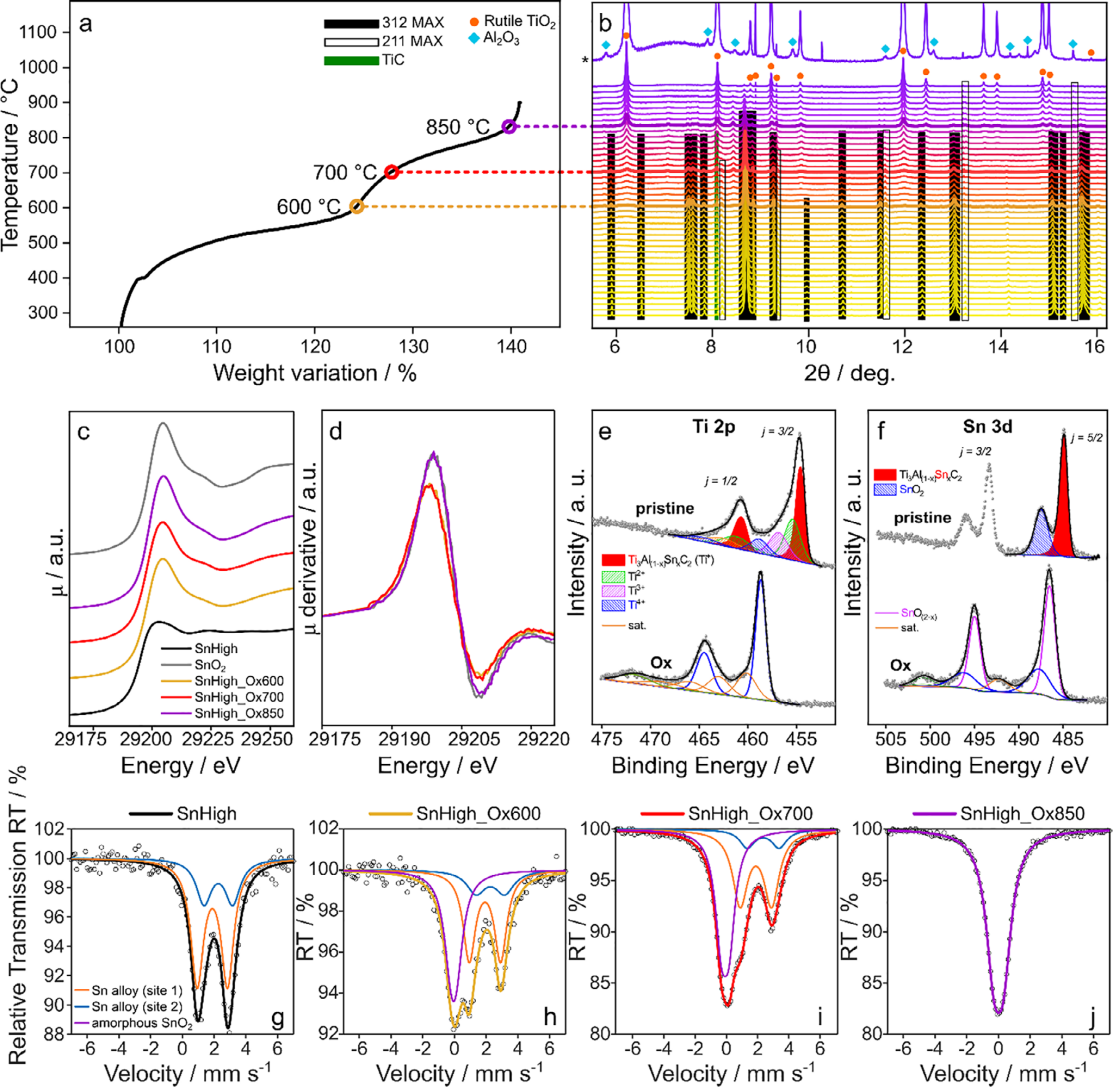
TGA profile for the pristine SnHigh sample heated in air in the temperature range of 300–900 °C (a). In situ XRD patterns of the SnHigh sample heated in air flow in an open capillary in the 300–1000 °C temperature range. The PDF cards of the phases are Ti_3_AlC_2_ 98‐016‐0572, Ti_2_AlC 98‐060‐6270, TiC 98‐065‐8339, rutile TiO_2_ 98‐003‐9171, corundum Al_2_O_3_ 98‐016‐0607. The pattern marked with * is collected at 1000 °C with a zoom on the y‐axis to clearly show the small reflections from alumina (b). Sn K‐edge XANES spectra of SnHigh, SnHigh_Ox600, SnHigh_Ox700, and SnHigh_Ox850. The spectrum of SnO_2_ is shown as a reference (c). First derivative of the XANES spectra of SnHigh_Ox600, SnHigh_Ox700, and SnHigh_Ox850 compared to SnO_2_, showing that the spectra of SnHigh_Ox600 and SnHigh_Ox700 are shifted to lower energies with respect to SnHigh_Ox850 and SnO_2_ (d). Ti 2p (e) and Sn 3d (f) XPS spectra of SnHigh and SnHigh_Ox700 (the signal is identical for the three oxidized samples). ^119^Sn Mössbauer spectra of the investigated samples: SnHigh (g), SnHigh_Ox600 (h), SnHigh_Ox700 (i), and SnHigh_Ox850 (j).

The partial oxidation of the SnHigh was studied using a combination of techniques (**Figure** **1**), which allowed both the determination of the extent of the oxidation reaction as a function of treatment temperature and the quantification of the different phases. Supposing that the MAX phase at the end of the oxidation treatment has the same composition as the pristine one, the oxidation reaction can be written as:

(3) $$\begin{array}{cc} & Ti_{3}Al_{0.3}Sn_{0.7}C_{2}+5.925x\;O_{2}\to \left(1-x\right)Ti_{3}Al_{0.3}Sn_{0.7}C_{2}\\ & +3x\;TiO_{2}+0.15x\;Al_{2}O_{3}+0.7x\;SnO_{2}+2x\;CO_{2} \end{array}$$

In this equation, *x* measures the extent of oxidation. For a more generic discussion considering the tin content as a second variable, see Equation S1 and Figure S1 (Supporting Information), and discussion therein.

The Thermogravimetric Analysis (TGA) profile (Figure 1a) shows the characteristic increase in weight caused by the incorporation of oxygen due to the formation of TiO_2_, SnO_2_, and Al_2_O_3_ despite the loss of carbon as CO_2_. It is well known that the replacement of Al by Sn in the MAX phase reduces its oxidation resistance, shifts the onset of weight gain to lower temperatures, and increases its kinetics.^[^ ^43^, ^50^, ^51^, ^52^, ^53^ ^]^ The reaction proceeds in two steps, attributed to the oxidation of Ti and Sn, leading to the formation of rutile/cassiterite‐like structures, and the subsequent complete oxidation of Sn and Al to their respective oxides. This is also confirmed by in situ XRD during the heating process. The analysis of the room temperature pattern confirms the presence of the pristine 312 MAX phase as the main contribution, with experimental composition slightly different from the expected one, Ti_3_Al_0.4_Sn_0.6_C_2_. A fraction of a 211 MAX phase (e.g., Ti_2_AC, with A = Al, Sn) is also detected (6 wt%) together with a small fraction of TiC (<1 wt%); full details are reported in SI, Table S2 (Supporting Information). Minor amounts of the 211 MAX phase are commonly observed during the preparation of the Al/Sn 312 MAX phase, and their presence is largely affected by the experimental parameters and synthesis protocol. The presence of a doped 211 MAX phase is not detrimental to the final application since its thermal behavior is similar to that of the 312 MAX phase, as previously reported.^[^ ^43^ ^]^ TiC is also one of the most common impurities obtained during the synthesis of MAX phases, and, contrary to the 211 MAX phase, its presence is not beneficial as it is electrochemically inactive and thermally inert. Nevertheless, an advantage of Sn doping of Ti_3_AlC_2_ is the minimization of the TiC amount, as confirmed by the comparison with previous results on the synthesis of pure Ti_3_AlC_2_.^[^ ^43^, ^54^ ^]^

The evolution of the XRD patterns with increasing temperature (Figure 1b), in agreement with TGA, shows that the sample is stable up to 300 °C, whereas a continuous subtle evolution of the reflections of the 312 and 211 MAX phases is detected above 400 °C, with a decrease of their intensities. Additional new peaks start appearing only above ≈550 °C, initially extremely broadened, their shape strongly evolving with increasing temperature. At 1000 °C, the sharp reflections of well‐crystallized rutile/cassiterite and alumina can be clearly identified, while the 312 and 211 MAX phase reflections virtually disappear. In summary, in situ XRD rationalizes the slopes and steps observed by TGA, globally confirming the gradual oxidation of the pristine material to crystalline (Ti/Sn)O_2_ and Al_2_O_3_ up to 1000 °C.

**Figure 2:**
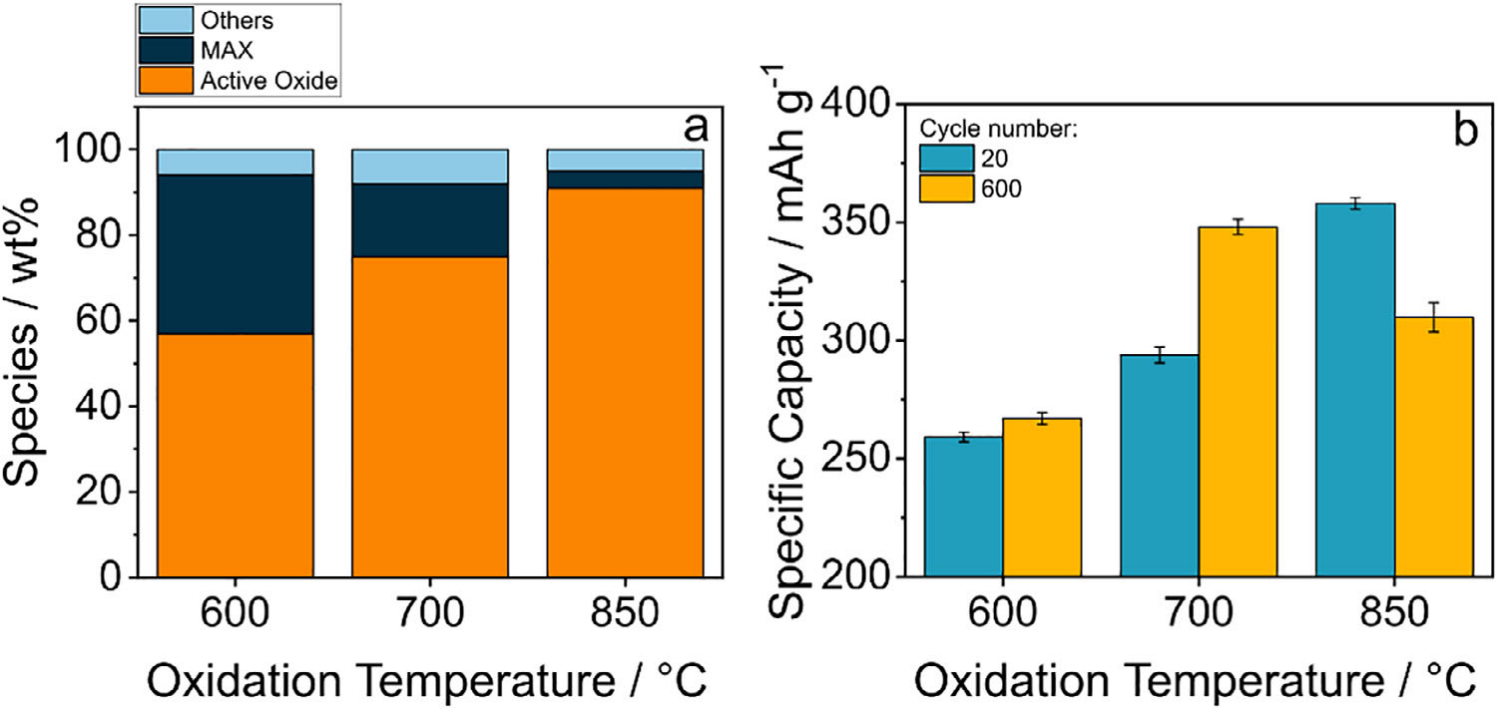
Evolution of phase composition (a) and specific capacity (b) with the oxidation temperature of the pristine MAX phase. In panel (a), based on the data of Table S1 (Supporting Information), Ti and Sn oxides are represented together under the label "Active Oxide" (orange bar), while the inactive species (mainly Al_2_O_3_ and carbon) but MAX are labelled as "Others" (light blue bar). Panel b shows the capacity values at the beginning (10 cycles mean around cycle 20) and in the middle (10 cycles mean around cycle 600) at 100 mA g^−1^ of the long cycling of SnHigh_Ox600, SnHigh_Ox700, and SnHigh_Ox850 reported in Figure S5 (Supporting Information).

This trend in the mass fractions of the components is in line with that of the same ones obtainable by combining Equation 1 with the mass increase data evaluated by weighing the sample after heat treatment, which is presented in **Figure** **2**a (more details in the dedicated section of the Table S1, Supporting Information). As the temperature of heat treatments increases, the relative amount of electro‐active oxides (SnO_2_, TiO_2,_ and their possible solid solutions) increases at the expense of the MAX phase. For the SnHigh_Ox700 sample, these values closely resemble those obtained by Rietveld refinement of the corresponding XRD pattern (cf. Table S2, Supporting Information), with a combined mass of active oxides accounting for 72.5 wt% of the total mass against the 75 wt% as calculated from Equation 1 and mass gain data. The discrepancy between the values, aside from the intrinsic uncertainties of the two methods, is probably mostly due to the differences in the experiments themselves, as XRD was carried out without the isothermal step, which is conversely present in each thermal treatment.

Given the partially amorphous nature of the intermediate species detected by XRD below 1000 °C, three samples, SnHigh_Ox600, SnHigh_Ox700, and SnHigh_Ox850 were studied by ex situ XAS, XPS, and ^119^Sn Mössbauer spectroscopy. Furthermore, the Rietveld analysis of the diffraction patterns of pristine and oxidized MAX is reported in Figure S2 and Table S3 (Supporting Information). The small particle size (often nanometers, as discussed in more detail in the section on TEM measurements) and the likely percentage of amorphous compounds resulting from the heat treatment make the refinement results incomparable with those of gravimetric analysis for the SnHigh_Ox600 sample. For the other two samples, the Rietveld result and the gravimetric measurements (Table S1, Supporting Information) are in good agreement, although in the following discussion, the compositional values from Table S1 (Supporting Information) will be used, as they are independent of the structure of the resulting phases.

The Sn K‐edge XAS spectra of SnHigh_Ox600, SnHigh_Ox700, and SnHigh_Ox850 are shown in Figure 1c, together with the reference spectra of the pristine MAX phase (black) and SnO_2_ cassiterite (grey). Overall, the spectra of the thermally treated samples show a close similarity to that of SnO_2_, indicating that tin has an oxidation state close to Sn(IV) in cassiterite. Considering that cassiterite and TiO_2_ (as rutile) are isostructural, it is reasonable to assume that tin is involved in a solid solution with TiO_2_ (more details in the section dedicated to the XRD analysis). However, while the spectra of SnHigh_Ox850 and SnO_2_ are nearly identical, the spectra of SnHigh_Ox600 and SnHigh_Ox700 are slightly shifted to lower energies with respect to the reference, indicating a slightly lower average oxidation state. The shift is more evident in the first derivative (see Figure 1d), while the intensity of the white line is much lower for SnHigh_Ox600 and SnHigh_Ox700 with respect to SnO_2_. These results suggest that part of the tin is still incorporated in the MAX phase up to 700 °C. Indeed, the spectra of SnHigh_Ox600 and SnHigh_Ox700 can be reproduced as linear combinations of the spectra of SnO_2_ and of the pristine MAX phase (cf. SI). Conversely, as mentioned above, in the SnHigh_Ox850 sample, all tin is found as Sn(IV) in a rutile‐type phase.

Further information about the oxidation process can be obtained by XPS. The Ti 2p spectra of pristine SnHigh and SnHigh_Ox700 are compared in Figure 1e. In the Ti 2p region, characterized by a series of spin‐orbit doublets (J = 3/2, 1/2) with an energy separation ΔE_so_ = 5.8 eV, the full‐width at half‐maximum of the two components used in the curve‐fitting was larger for the j = ½ component, in order to account for the known broadening due to the Coster‐Kronig effect in Ti 2p ionization.^[^ ^55^ ^]^ In the pristine MAX sample, each doublet can be assigned to a different chemical state of Ti (see Table S4, Supporting Information). The lowest BE component (red) is assigned to Ti(I), displaying metallic character within the pure MAX phase, closely matching the position and shape asymmetry found in parental TiC species.^[^ ^56^ ^]^ The following components at increasing BE can be assigned to Ti(II), Ti(III), and Ti(IV) states, probably associated with Ti oxides^[^ ^57^, ^58^, ^59^, ^60^ ^]^ formed upon contact with air. In the thermally treated SnHigh_Ox700 sample, a complete oxidation of Ti is observed, since only the contribution from TiO_2_ is visible (blue curves). The high intensity of the signal allows the observation of the typical weak charge‐transfer satellites of TiO_2_ (yellow curves).^[^ ^61^, ^62^, ^63^, ^64^ ^]^

In the Sn 3d spectrum of pristine SnHigh (Figure 1f), two different spin‐orbit doublets (J = 5/2, 3/2) with an energy separation of ΔE_so_ = 8.4 eV can be assigned two main Sn oxidation states (cf. Table S4, Supporting Information), with the predominance of a low BE component (magenta curve, J = 5/2 at 484.92 eV) attributed to metallic tin (Sn^0^), as expected for the pure MAX phase.^[^ ^23^ ^]^ The additional Sn 3d_5/2_ broader component at 487.45 eV is compatible with the presence of SnO_2_ (blue curve).^[^ ^23^, ^65^, ^66^ ^]^ In the case of the annealed sample SnHigh_Ox700, a single major feature at an intermediate BE value between pure SnO^[^ ^67^, ^68^ ^]^ and SnO_2_ is observed,^[^ ^65^, ^66^ ^]^ probably due to the formation of sub‐stoichiometric Sn(IV) oxide, here indicated as SnO_2‐x_ (magenta curve, 486.57 eV).^[^ ^69^, ^70^, ^71^, ^72^, ^73^ ^]^ Two additional components, however, were necessary for an accurate curve‐fitting: the first one may be associated to minor amounts of SnO_2_ (blue curve), while the other, behaving as a spin‐orbit doublet (yellow curve), might be a “satellite” (charge‐transfer or *shake‐up*) line associated to the SnO_2‐x_ component. This feature has never been reported in the literature for tin compounds to the best of our knowledge and could therefore be peculiar to the chemical environment of these mixed Sn(II)/Sn(IV) species in the oxidized MAX phase.

The C 1s spectra of the MAX phase samples (Figure S4a,b, Supporting Information) exhibit a sequence of peaks: the lower BE one in pristine SnHigh_Ox700 (Figure S4a, Supporting Information) is associated with the pure MAX phase (component at 281.8 eV), where C has a metallic carbide character.^[^ ^60^ ^]^ The more intense features at higher BE can be attributed to environmental adventitious carbon species, such as aliphatic and graphitic contaminants (red curve, 285.00 eV), to oxidized carbon contaminants containing C‐OH (blue curve), and to COOH (orange curve) groups (cf. Table S4, Supporting Information). Upon annealing, the low BE energy carbide component is no longer visible, as expected according to the formation of CO_2_ upon oxidation, while the contributions from organic carbon impurities are still observed.

Finally, the BE of the Al 2p spectra of the pristine and the annealed MAX phase (Figure S4c, Supporting Information,d), both fitted to a single unresolved spin‐orbit doublet (J = 3/2, 1/2) with an energy separation of ΔE_so_ = 0.4 eV,^[^ ^56^ ^]^ was found to change from 72.45 to 74.20 eV, respectively. These positions are compatible with the presence of metallic Al, characteristic of the pristine MAX phase, and Al_2_O_3_, resulting from the oxidation process.^[^ ^56^, ^74^, ^75^ ^]^

To better understand the evolution of the tin species during thermal oxidation, ^119^Sn Mössbauer spectra were measured for samples SnHigh, SnHigh_Ox600, SnHigh_Ox700, and SnHigh_Ox850 (cf. Table S5 (Supporting Information) for the fitted hyperfine parameters). The spectrum of pristine SnHigh (Figure 1g) shows an asymmetric quadrupole doublet, with average isomer shift (δ) and quadrupole splitting (Δ) of 2.0 and 1.9 mm s^−1^, respectively, in the typical range of tin alloys.^[^ ^76^ ^]^ This doublet can be fitted with (at least) two slightly shifted subspectra, probably representing slightly different chemical environments around the tin centers, e.g., different numbers of Sn and Al neighbors.

In the spectrum of sample SnHigh_Ox600 (Figure 1h), the quadrupole doublet representing the MAX phase is accompanied by a second component with a slightly negative δ and unresolved Δ, which can be attributed to Sn(IV) oxide. It is interesting to notice that such a δ value, commonly observed for Sn(IV) dissolved in glasses or other oxide mixtures, as well as in amorphous oxides, differs significantly from the typical one of crystalline cassiterite SnO_2_ (+0.02 mm s^−1^), and may indicate the formation of a mixed Ti‐Sn oxide phase.^[^ ^76^ ^]^ The relative intensity of this component reaches 38% of the resonance area, which can only be taken as a semi‐quantitative evaluation of the oxidized tin amount, as the Lamb‐Mössbauer factor of the MAX phase at room temperature is unknown. Finally, the ratio between the two components used to fit the MAX phase contribution is similar to that of pristine SnHigh.

The spectrum of sample SnHigh_Ox700 is similar to that of SnHigh_Ox600 (Figure 1i), with the presence of the same tin species, but with a significant increase in Sn(IV) oxide contribution from 38 to 47%, confirming that a larger amount of tin is oxidized. Interestingly, compared to the previous samples, the relative intensity of the two components used to fit the MAX phase is modified. This might indicate that, as oxidation proceeds, one of the two sites has a greater tendency to oxidize.

Finally, the spectrum of sample SnHigh_Ox850 (Figure 1j) shows only the contribution of Sn(IV) oxide in the form of cassiterite, with a δ value of 0.02 mm s^−1^, indicating that the Sn‐containing portion of the MAX phase is completely oxidized, in full agreement with the XAS data.^[^ ^76^ ^]^ The residual MAX phase detected by XRD at the end of the annealing process, therefore, represents the Sn‐free fraction, which is more resistant at high temperature.

### 2.2 Composition and Electrochemical Properties of the Annealed MAX Phases

The formation of different amounts of TiO_2_, SnO_2,_ and their solid solutions seems to be a crucial parameter for optimizing the electrochemical performance, as it could be verified by testing SnHigh_Ox600, SnHigh_Ox700, and SnHigh_Ox850 in half cells versus Li metal (Figure 2b; Figure S5, Supporting Information).^[^ ^77^ ^]^ The cells were submitted to 10 cycles at 50 mA g^−1^, followed by 300 cycles at 100 mA g^−1^, repeated three times up to 930 cycles. Theoretically, the higher the temperature at which the MAX phase is treated, the more abundant the oxide formation (Figure 2a), resulting in larger amounts of electrochemically active species participating in conversion and alloying processes, and thus leading to a higher specific capacity. However, after ≈50 cycles, the sample SnHigh_Ox850 drastically loses capacity. This drop in performance can be correlated to the electrode breakdown caused by the volume change occurring during the (de)alloying processes.^[^ ^16^ ^]^ The other two samples, however, do not show this peculiar behavior, indicating that the residual presence of the MAX phase helps the electrode to withstand the mechanical stress caused by volume variation during cycling.

The results of the long cycling analysis of the three samples are summarized in **Table** **1**. By combining the theoretical specific capacities of SnO_2_ (alloying only) and TiO_2_ (≈300 mAh g^−1^) with the compositional data in Table S1 (Supporting Information), the expected capacities of the oxidized phases are ≈250, 335, and 400 mAh g^−1^ for SnHigh_Ox600, SnHigh_Ox700, and SnHigh_Ox850, respectively. The experimental results in Table 1 show excellent consistency with these estimates. However, while SnHigh_Ox850 achieves the highest first‐cycle specific capacity (402 mAh g^−1^), its lower I cycle reversibility (52.4%) and low efficiency (<99%) suggest performance trade‐offs. Conversely, SnHigh_Ox600 shows better I cycle reversibility (57.4%) but significantly lower capacity (286 mAh g^−1^). The tradeoff between electrochemical performance and the sample composition is clearly illustrated in Figure 2. The MAX phase plays a pivotal role in the electrode, contributing not only to efficient electron transport but also to preserving its structural integrity during cycling. Although electrochemically inactive, its presence is essential to prevent electrode degradation; however, this same inertness leads to a reduction in the overall specific capacity of the composite.

**Table 1:** Electrochemical performance of the three samples during long cycling measurements. The capacity retention was evaluated between the first and 100th cycle at 100 mA g^−1^, while the mean specific capacities and coulombic efficiency refer to the values after stabilization (i.e., average of 10 cycles around cycle 600). The specific capacities are calculated considering the anodic process (de‐lithiation).

Sample	Specific capacity 1st cycle/mAh g^−1^	Reversibility at 1st cycle/%	Mean specific capacity at 100 mA g^−1^/mAh g^−1^	Mean coulombic efficiency at 100 mA g^−1^/%
SnHigh_Ox600	286	57.4	257 ± 13	99.2 ± 1.7
SnHigh_Ox700	347	54.1	330 ± 18	99.2 ± 1.9
SnHigh_Ox850	402	52.4	304 ± 19	98.9 ± 2.7

SnHigh_Ox700, with a good first‐cycle capacity (347 mAh g^−1^), and a stable mean coulombic efficiency (99.2% ± 1.9), strikes the most balanced performance, making it the most promising candidate for reliable long‐term use. It was therefore selected for a thorough structural, morphological, and electrochemical characterization.

### 2.3 Structural, Morphological, and Electrochemical Properties of SnHigh_Ox700

**Figure 3:**
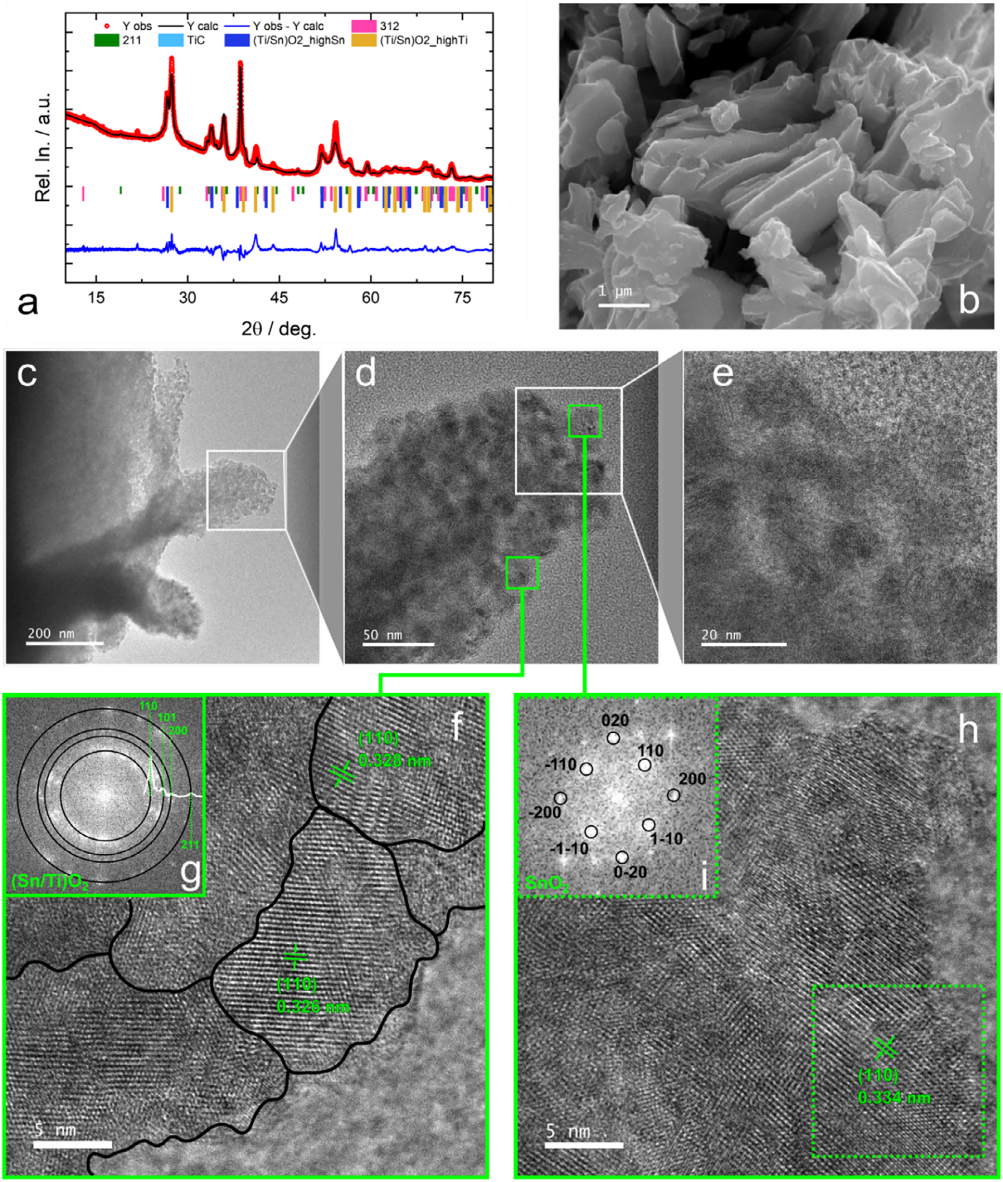
SnHigh_Ox700 structural and morphological characterization. Rietveld refinement for the XRD data (Rwp 7.15; Chi2 10.32) (a). SEM image at medium magnification (15kx) (b). Sequence of TEM images with increasing magnification (c–e), HRTEM images of two different spots (f, h) with their relative FFT‐derived diffraction images (g, h). The FFT‐derived diffraction pattern of the Sn/TiO_2_ phase (PDF Card – 01‐070‐4408) is evidenced.

The XRD pattern of SnHigh_Ox700 was refined using the Rietveld method to determine the phase composition of the material (**Figure** **3**a). The main crystalline phase in SnHigh_Ox700 is (Ti/Sn)O_2_ (≈77%), whereas the 312 and 211 MAX phases constitute together ≈23% of the composite. The refinements were carried out by using two populations of (Ti/Sn)O_2_ characterized by different cell parameters and crystallite size (cf. Table S2, Supporting Information), suggesting that two different Ti/Sn oxide compositions are obtained.

The morphological analysis of SnHigh_Ox700, carried out by SEM (Figure 3b), shows a broad distribution of grain sizes in the 1‐5 µm range, the biggest grains being secondary aggregate structures. The surface of each MAX grain is covered by a dense layer of nanometric particles, and such texture is even clearer in the TEM analysis (Figure 3c): the micrometric grain is completely covered by nanometric crystalline structures with a size of the order of 10–20 nm (Figure 3d,e). Such nanometric domains, evidenced by a black line in Figure 3f, were extensively analyzed by HRTEM with magnifications between 500 kx and 1.5 Mx to get as much information as possible about their nature (Figure S6, Supporting Information). The d‐spacing of the crystalline structures was obtained by FFT analysis of the whole HRTEM images (Figures 3g,i). In Figure 3g, obtained by FFT analysis of the whole Figure 3f, several randomly oriented domains are detected, whereas a single domain is identified in Figure 3i, obtained by FFT analysis of the square region marked with the dotted green line in Figure 3h. Such patterns can be attributed to the rutile‐like phase family, including TiO_2_, SnO_2,_ and their solid solutions, identified by (110) spacings varying between those of pure TiO_2_ (0.324 nm) and SnO_2_ (0.335 nm), in agreement with previous findings.^[^ ^43^ ^]^ Variable Sn/Ti ratios were found in different regions of the sample, as shown in Figure S6a (Supporting Information).

**Figure 4:**
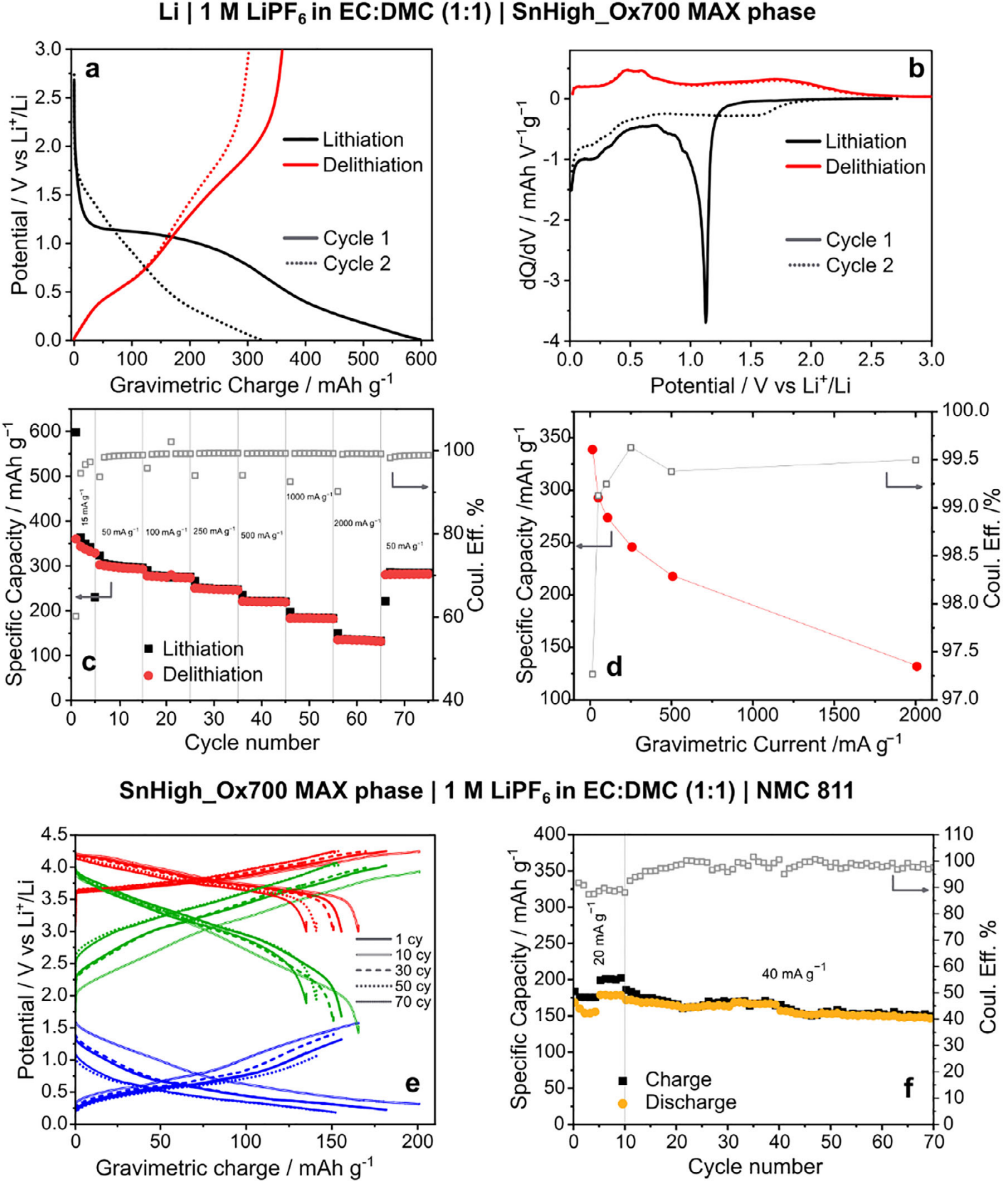
Electrochemical characterization of SnHigh_Ox700: galvanostatic profiles versus Li metal, cycle 1 and 2 (a), with correlated differential capacity (b); rate capability versus Li metal (c), and average values of capacity and efficiency versus gravimetric current (d). Full cell cycling versus NMC 811 (positive and working electrode, WE): galvanostatic profiles of WE, CE, and WE–CE taken at cycles 1, 10, 30, 50, and 70 (e); long‐cycling tests of the full cell (f). Gravimetric charge and specific capacity are calculated based on the mass of active species of the MAX phase for the half‐cell analyses, while the mass of NMC has been used for the full cell measurements.

The electrochemical properties of SnHigh_Ox700 were studied in detail in half‐cell versus Li metal. The corresponding galvanostatic profile and its derivative during the first two cycles at 15 mA g^−1^, the rate capability, and the capacity retention are shown in **Figure** **4**a,b. The long plateau observed at 1.1–1.2 V in the first cycle (main peak in the derivative curve), associated with the conversion of SnO_2_ to metallic Sn,^[^ ^24^ ^]^ significantly shortens during the second cycle, confirming the predominantly irreversible nature of this reaction. Furthermore, the change in slope of the galvanostatic curve at 0.4–0.5 V in lithiation (and corresponding peak in the differential capacity plot), associated with the Sn alloying reaction, remains unchanged between the two cycles, proving the good reversibility of this process.^[^ ^24^ ^]^ During the second lithiation, a minor contribution to the capacity is observed starting from 1.7 V, possibly related to the lithiation of rutile. The first cycle specific capacity in delithiation is of 360 mAh g^−1^, with a reversibility of 60%, while during the second cycle it decreases to 303 mAh g^−1^, with a reversibility of 94%. The rate capability test (Figure 4c,d) shows a reversible specific capacity decreasing from 294 to 291 mAh g^−1^at 50 mA g^−1^, gradually decreasing to 133 ± 1 mAh g^−1^ by increasing the current up to 2000 mA g^−1^. The coulombic efficiency, at 96.1% during the first cycles, gradually increases during cycling and eventually stabilizes at 95.5% in the last cycles. This excellent behavior at high currents is one of the most relevant features of this material, being significantly higher than the commonly obtained capacity of commercial graphite anodes.^[^ ^78^ ^]^ Furthermore, the composite proposed in this work also offers advantages over similar materials (based on titanium and tin and/or incorporated in matrices to stabilize them during cycling). A thorough comparison between these samples is reported in Table S6 (Supporting Information). Although it performs worse than, for example, tin‐based alloys, the composite proposed here has clear benefits in capacity retention and coulombic efficiency, even when cycled at low currents.^[^ ^79^, ^80^, ^81^, ^82^, ^83^ ^]^ The proposed sample is also top tier in terms of coulombic efficiency, even when compared to SnO_2_‐based systems incorporated in carbon/MXene matrices, a symptom of the excellent ability of the system to drastically reduce the notorious volumetric caries typical of insertion/alloying systems.^[^ ^20^, ^84^, ^85^, ^86^, ^87^, ^88^, ^89^ ^]^

Negative electrodes based on SnHigh_Ox700 were further tested in a Hohsen full‐cell setup versus commercial NMC811 (Figure 4e,f). The negative electrode was prelithiated before being inserted into the cell to optimize its performance and avoid the irreversible loss of capacity due to the conversion of SnO_2_. The cell shows a mean specific capacity of 157 ± 12 mAh g^−1^ with a mean coulombic efficiency of 98.5 ± 1.8% and a mean round trip efficiency of 95.6 ± 1.5%, at a current density value of C/5 for NMC811 (theoretical capacity 200 mAh g^−1^). It should be noticed that, under these conditions, the full capacity of the negative electrode was not exploited, the NMC positive electrode being the one limiting the capacity of the full cell. Improved performance, therefore, is expected by optimizing the capacity balance between the two electrodes.

### 2.4 Electrochemical Mechanism of SnHigh_Ox700

To shed light on the electronic and structural changes occurring in the SnHigh_Ox700 sample upon lithiation, in situ Sn K‐edge XAS was carried out on a working cell during the first electrochemical cycle (Figure 7a). Upon lithiation, the spectra rapidly shift toward lower energies, while the white line intensity decreases drastically, indicating the reduction of Sn(IV) to Sn(0). At ca. 0.45 V versus Li^+^/Li, well before the end of lithiation, all tin is in the metallic state. However, the spectrum recorded at 0.4 V (Figure S7, Supporting Information, blue line) does not fully match that of the reference tin foil. Moreover, the spectrum at 0.4 V, roughly in the middle of the lithiation process, displays significant differences from that recorded at 0.01 V, i.e., at the end of lithiation (cf. Figure S7, Supporting Information), which slightly shifted to higher energies. Even though a shift to higher energy commonly indicates oxidation, this hypothesis can be safely excluded at such a low potential. In light of previous studies on SnO_2_‐based anodes for LIBs, this modification is rather attributed to the formation of Sn‐Li alloys, as a consequence of the charge‐transfer occurring between the two atomic species.^[^ ^90^, ^91^ ^]^ Considering that even at 0.4 V the spectrum does not coincide with that of tin metal, most probably the alloying process starts before the complete reduction of Sn(IV) to Sn(0).

Strong differences between the spectra at 0.4 and 0.01 V are also observed in the EXAFS region (Figure S7, Supporting Information). In fact, while the spectrum at 0.4 V exhibits roughly the same oscillations as the tin foil spectrum, even though notably damped, the spectrum at 0.01 V shows an almost flat EXAFS signal. This indicates the presence of a very limited short‐range order, compatible with the formation of amorphous Li‐Sn species. Such a decrease in intensity in the EXAFS region is paired by the evolution of the corresponding FTs (Figure S8, Supporting Information), where the peak at ca. 2 Å in the pristine compound, corresponding to the first Sn‐O neighboring shell of SnO_2_, decreases significantly in intensity and disappears completely below 0.4 V, in agreement with the full reduction of tin observed by XANES. Interestingly, the decrease of this peak is not accompanied by a corresponding increase of the Sn‐Sn shell, expected at ca. 3 Å for metallic Sn, suggesting the formation of zerovalent tin species with limited short‐range order. With the formation of lithium‐tin alloys, nevertheless, the intensity of the Sn‐Sn should decrease again, as already observed also for SnO_2_ anodes.^[^ ^90^ ^]^ Indeed, below 0.3 V, no peak above the background noise can be clearly distinguished in the FT.

**Figure 5:**
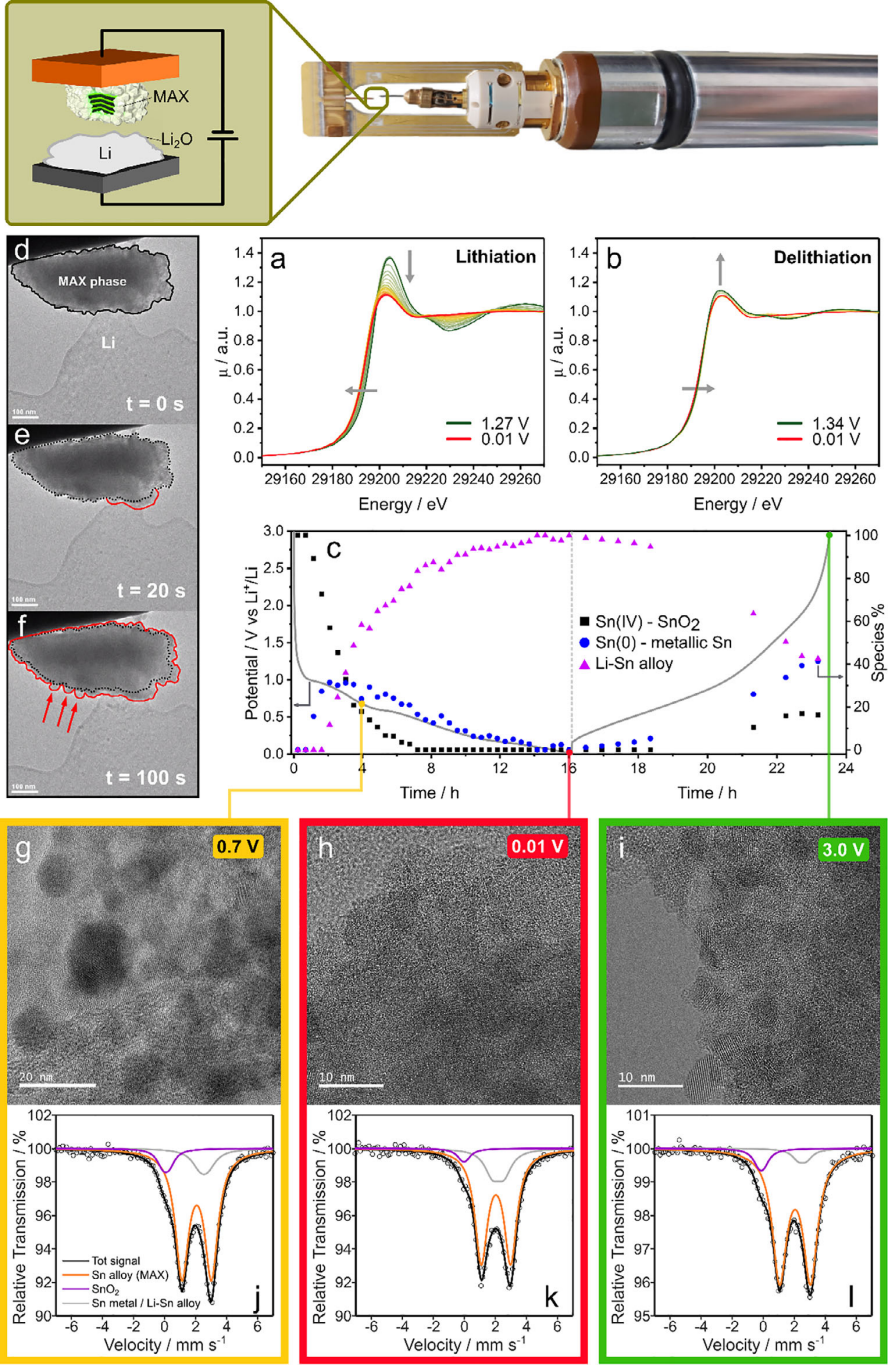
In situ Sn K‐edge XAS spectra recorded during the first lithiation (a) and delithiation (b): beginning (1.27 V) and end (0.01 V) of lithiation are highlighted by a dark green and a red line, respectively (a); end of delithiation (1.34 V) is marked by a dark green line (b). Evolution of the concentration of the Sn species during the lithiation/delithiation processes (c). In situ TEM images of a SnHigh_Ox700 grain (Particle 1) upon lithiation: no contact and no bias (d); 20 s after applying bias (e); 100 s after applying bias (f). HRTEM images of the sample stopped at 0.7 V during the first lithiation (g), at 0.01 V at the end of the first lithiation (h), and at 3 V at the end of the first delithiation (i). ^119^Sn Mössbauer spectra of SnHigh_Ox700 cycled electrodes at different potentials: 0.7 (j), 0.01 (k), and 3 V (l); the different species are indicated with different colors: Sn in MAX phase in orange, Sn(IV) oxide in purple, metallic tin and lithium‐tin alloy(s) in grey.

A semiquantitative evaluation of the Sn K‐edge XAS spectra was carried out by Linear Combination Fitting starting from the spectra of reference compounds to follow the evolution of the different species formed upon lithiation. As reference spectra of Li‐Sn alloys are not available in the literature, the last spectrum acquired upon lithiation at 0.01 V was used as the reference for the Li‐Sn alloy. This assumption, which implies that all tin is fully lithiated at the end of the process, does not consider that different alloy compositions can be formed during lithiation, which may display slightly different spectral signatures. To partially overcome this issue, a LCF analysis was carried out on the derivative spectra, which are particularly sensitive to possible energy shifts related to the charge‐transfer occurring between Sn and Li, using the spectrum of the pristine sample, the Sn foil, and the spectrum at 0.01 V representing the final Li‐Sn alloy. The possible formation of SnO was neglected since, even though the formation of transient Sn^2+^ species cannot be excluded, especially at the beginning of the lithiation, they would rapidly disappear, as Sn is promptly reduced to the metallic state during the first few spectra. The results of the LCF (**Figure** **5**c) show that, during the first plateau between 1.0 and 0.6 V, Sn(IV) is reduced to tin metal, as expected for the conversion reaction. However, while the conversion is still ongoing, the Sn‐Li alloy starts to form, suggesting that both the conversion and the alloying mechanism proceed simultaneously for a while. During the second plateau, the remaining Sn(IV) is completely reduced to Sn(0), while the amount of the Li‐Sn alloy increases at the expense of metallic Sn. As the potential decreases further, the amount of Li‐Sn alloy increases, and reaches its maximum concentration at the end of the lithiation. During the following delithiation (Figure 5b), neither the white line intensity nor the edge energy position is restored, indicating a high degree of irreversibility. The LCF shows that metallic Sn(0) is partially reformed because of the dealloying reaction. Moreover, above ca. 1 V, part of the tin is re‐oxidized to Sn(IV) species and, at the end of delithiation (2.3 V), the XAS spectrum can be obtained by the LCF of Sn metal, Sn‐Li alloy, and of the pristine sample with relative concentrations of 41(3)%, 43(4)%, 16(1)%, respectively.

The in situ XAS analysis was accompanied by an extensive in situ and ex situ TEM study. Ex situ HRTEM measurements were carried out on SnHigh_Ox700 electrodes cycled at 0.7 V during the first lithiation, at 0.01 V at the end of the first lithiation, and at 3 V at the end of the first complete cycle. Examples of the morphology of each state of charge are reported in Figure 5g–i, whereas the corresponding d‐spacing intensities obtained by 2D FFT of the same images are shown in Figures S9e, S11e, and S12e (Supporting Information). At the end of the first lithiation plateau at 0.7 V, in agreement with XAS results, agglomerates of spherical particles with sizes in the 10–20 nm range (Figure S9, Supporting Information) are observed. These particles can be identified as Sn metal, based on the 0.201 nm d‐spacing, corresponding to the (211) planes of metallic Sn (Figure S10, Supporting Information). The peaks associated with metallic Sn are rarely observed in the reconstructed diffraction patterns since the Sn particles are embedded in oxide matrices, which makes their lattice spacings difficult to focalize (cf. Figure 5g). Conversely, the predominant peak in Figure S9e (Supporting Information) (see lines a‐d) is identified with Li_4_Ti_5_O_12_ (LTO), which may be produced during the lithiation/conversion of the Ti_1‐x_Sn_x_O_2_ solid solution. At 0.01 V (Figure 5h; Figure S11, Supporting Information), the morphology of the sample is so confused and blurred that it is difficult to clearly discern neat phases. This is confirmed by the diffraction intensity patterns (Figure S11e, Supporting Information), where a large bump centered at 0.313 nm dominates the d‐spacings, possibly resulting from the convolution of broad signals belonging to LiSn, along with peaks ≈0.206 and 0.221 nm, probably associated with β‐LiSn. Nevertheless, the observed particles are so small that it is difficult to identify the coherence length of the crystalline domains, and the contribution of other unknown phases cannot be excluded. At 3 V (Figure 5i; Figure S12, Supporting Information), small crystalline particles with sizes in the range 5–10 nm are observed. The domains are sensibly smaller than in the pristine material, probably due to the degradation caused by volume change upon lithiation and delithiation. The reconstructed diffraction patterns are more difficult to interpret, as such small domains produce broader and more confusing peaks. Moreover, several phases seem to be present, including cassiterite SnO_2_ and α‐SnO_2_ (peaks at 0.335 and 0.298 nm), as well as TiO_2_ (peak at 0.324 nm). The remaining peaks could be associated with SnO_2_ and Sn metal (Figure S12e, Supporting Information), already observed by XAS.

The volumetric expansion of the material was followed by in situ TEM during lithiation (cf. Particle 1 in Figure 5d–f; Particle 2 in Figure S13 (Supporting Information) and Videos in Supplementary Material). In these experiments, a particle of SnHigh_Ox700 attached to a copper wire is put in contact with metallic lithium deposited on a tungsten wire inside a two‐electrode Dual‐Probe in situ holder, the natural Li_2_O thin passivation layer on Li metal acting as a solid electrolyte. For both experiments, we increased the bias in 0.1 increments up to 1.5 V, pausing for 10 min at each step to allow for slow reaction progress and determine the optimized value. An optimal bias of 1.5 V applied to the system for 20 s induced swelling of the oxide particles near the contact point, while the core remained unchanged. After ≈100 s, the whole external nanoparticle shell is swollen. An estimation of the particle swelling can be obtained by the analysis of the image frames during the process: the pristine area of Particle 1 (11.6 × 10^4^ nm^2^) and Particle 2 (1.6 × 10^4^ nm^2^) are increased by 20% and 34% after 100 s, respectively. These values are consistent with the expected volume expansion of 300/400% of SnO_2_ during lithiation, as this species covers the surface of a non‐reacting MAX phase core.

The evolution of the tin species during cycling was also studied by ex situ ^119^Sn Mössbauer spectroscopy. To do this, free‐standing electrodes of SnHigh_Ox700 were prepared and cycled versus lithium at the same potentials investigated with ex situ TEM: 0.7 V during the first lithiation, 0.01 V at the end of the first lithiation, and 3.0 V after the first delithiation. Compared to that of the pristine electrode at OCV (Figure 1i), the spectrum of the electrode lithiated at 0.7 V shows a decrease in intensity of the peak related to tin oxide, and the appearance of a new unresolved doublet with an IS of 2.5 mm s^−1^, in the typical range of β‐Sn metal^[^ ^76^ ^]^ (Figure 5j). The relative intensity of these spectral components, reported in **Table** **2**, cannot be directly related to the semiquantitative speciation of tin, as the Lamb‐Mössbauer factors of the different tin species at room temperature may differ significantly. In particular, the Lamb‐Mössbauer factor of tin metal is expected to be significantly lower than that of SnO_2_, leading to a substantial underestimation of the amount of Sn metal produced by the conversion reaction. However, assuming that the MAX phase does not react and its absolute amount does not change during lithiation, the ratio between the intensity of its doublet and that of Sn(IV) can be used to estimate the fraction of Sn(IV) converted to Sn metal. Based on this, the conversion of Sn(IV) approaches 90% at 0.7 V. In the spectrum of the electrode lithiated at 0.01 V (Figure 5k), the dominant unresolved doublet increases in intensity and shifts from 2.5 to 2.15 mm s^−1^. This shift can be associated with the formation of a Li‐Sn alloy with a composition close to Li_7_Sn_2_, usually obtained at the end of the lithiation of Sn anodes.^[^ ^92^ ^]^ Finally, in the spectrum obtained from the electrode re‐lithiated at 3.0 V, it is possible to verify the reversibility of the alloying‐dealloying process, with the main signal shifting back to 2.5 mm s^−1^ (Figure 5l), typical of the β‐Sn. It is interesting to notice that the relative amount of Sn(IV) does not vary sensibly in intensity with respect to the MAX phase compared to the spectrum at 0.7 V, which suggests that the conversion reaction is only very partially reversible. However, this strong irreversible behavior may be due to the experimental condition used for the preparation of the Mössbauer samples, which might hinder the reconversion process (cf. Figure S14, Supporting Information).

**Table 2:** Phase composition of the cycled electrodes obtained from the ^119^Sn Mössbauer spectra.

	Sn‐MAX / %	SnO_2_ / %	Metallic Sn / %	Li_7_Sn_2_ / %
OCV	54	46	–	–
0.7 V	80	8	12	
0.01 V	76	4	–	20
3 V	88	7	6	–

## 3 Conclusion

The controlled oxidation of the Sn‐containing MAX phase Ti_3_Al_0.3_Sn_0.7_C_2_ is a promising strategy to develop nanostructured composites with excellent electrochemical properties for use as negative electrodes in LIBs. The method relies on thermally oxidizing the MAX phase, resulting in the formation of titanium (TiO_2_) and tin (SnO_2_) oxides decorating the surface of residual MAX phase cores, which together create a composite material with distinctive features. In a study of the oxidation treatment at carefully regulated temperatures between 600 and 850 °C, the SnHigh_Ox700 sample, processed at 700 °C, emerges as the optimal material due to its balanced composition of oxides and residual MAX phase, enabling a superior trade‐off between specific capacity and reversibility.

The approach presented here offers two intrinsic advantages over the conventional mechanical mixing of the components: first, the direct growth of the active oxide on the MAX surface ensures more efficient charge transport, as electrons can move through the MAX and reach the Sn/TiO_2_ nanoparticles via intimate electrical contact, while the nanoparticles remain fully accessible to the electrolyte and ionic flux; second, the unique “quantum dot”‐like morphology allows the material to accommodate mechanical stresses associated with the volume changes typical of conversion/alloying materials such as SnO_2_. The residual highly conducting MAX phase is thus vital for stabilizing the electrode, effectively buffering volume fluctuations during repeated charge and discharge cycles while allowing an efficient electron percolation network. The SnHigh_Ox700 material demonstrated a good specific capacity of 290 mAh g^−1^ at 50 mA g^−1^, with a high reversibility of 99.1%, and an impressive rate capability under high charge rates, outperforming conventional graphite electrodes. This remarkable performance originates from the synergy between the active oxides and the structural stability imparted by the residual MAX phase.

Advanced in situ analyses (XAS, XRD, and TEM) were used to shed light on the electrochemical (de)lithiation mechanisms, and the contributions of the different active materials to the electrochemical behavior were carefully examined. While Al_2_O_3_ and the pristine, unreacted MAX phase do not provide significant capacity due to their low amount (Al_2_O_3_) or inert behavior (both), the thorough study of the role of SnO_2_ and its reaction mechanism revealed the partially reversible conversion of SnO_2_ to metallic Sn and the reversible alloying reaction between Sn and Li. During lithiation, nanoscale Li‐Sn alloy particles with limited short‐range order are formed, alongside volumetric expansion of the oxide nanoparticles, as confirmed by in situ TEM studies. On the other hand, the role of TiO_2_ is more difficult to assess: its intercalation capacity is lower than the conversion/alloying reactions of SnO_2_, and the Sn/TiO_x_ oxides exhibit a continuous, solid‐solution‐like composition. The only direct evidence of lithium titanate formation is provided by HRTEM, which shows that the structure of a sample cycled and stopped at 0.7 V appears compatible with lithiated titanium oxide.

Tests of SnHigh_Ox700 in full‐cell configuration, using NMC as the cathode, demonstrated excellent performance, highlighting its potential for real‐world applications. The nanostructured design achieved via controlled oxidation, combined with the unique composition of oxides and residual MAX phase, plays a critical role in improving stability and electrochemical performance.

## 4 Experimental Section

### Materials Synthesis

To synthesize the Ti_3_Al_0.3_Sn_0.7_C_2_ nominal composition, Ti, Al, Sn, and TiC powders were used as precursors in an atomic ratio of 1:0.4:0.7:1.85. The precursor powders were mixed in a Turbula mixer for 24 h. 12 grams of the mixed powders were then placed into a graphite die and positioned in the chamber of an SPS Dr. Sintermodel 925 (manufactured by Fuji, Japan). With the chamber pressure below 500 millibars, the powder mix was heated to 1350 at 80 °C min^−1^ and compressed mechanically up to 30 MPa for 30 min. After cooling at 80 °C, the sample was removed from the chamber. The resulting pellet was then sandblasted to clean the TiC‐rich surface and ground using a TiN‐coated tool to obtain the MAX phase powder.

### Thermal Treatment of MAX Phase

The MAX phase powders were evenly distributed in an alumina crucible and heated in an open tubular oven (Carbolite Gero). For the three oxidized samples, SnHigh_Ox600, SnHigh_Ox700, and SnHigh_Ox850, a temperature ramp of 7.5 °C min^−1^ was applied, reaching final temperatures of 600, 700, and 850 °C, respectively. All the ramps were followed by a dwell at their final temperature for 40 min.

### TGA

The investigated samples were analyzed by thermogravimetric analysis (TGA), with a PerkinElmer instrument, with a heating ramp of 7.5 °C min^−1^ in air flow, from 30 to 900 °C.

### Synchrotron XRD

High resolution high intensity XRD patterns were collected at the ID22 beamline, European Synchrotron Radiation Facility (ESRF, Grenoble, France), in transmission mode using a dedicated optical cell for electrochemical analysis. XRD patterns were collected continuously during the lithiation/delithiation process with a wavelength of 0.3543 Å.

### XAS

Sn K‐edge X‐ray absorption spectra (29200 eV) were acquired in fluorescence mode at the LISA beamline,^[^ ^93^ ^]^ (BM08, ESRF), using a Si(111) double‐crystal monochromator and Pt‐coated mirrors to suppress high‐order harmonics. Energy calibration was performed with a Sn metal foil. SnO and SnO_2_ were also measured as reference spectra in transmission mode. For these measurements, an appropriate amount of sample (calculated to give an absorption edge jump of 1 in logarithmic units) was mixed with pure cellulose and pressed into 1.3 cm diameter pellets. All data were measured at room temperature. The electrochemical cell used for in situ XAS measurements during electrochemical cycling was an ECC‐Opto‐Std test cell (EL‐CELL) equipped with ECC‐Opto Polymide windows.

XAS spectra extraction and normalization were performed using the ATHENA code, integrated in the software suite DEMETER.^[^ ^94^, ^95^ ^]^ The pre‐edge background was fitted with a straight line, and the post‐edge background with a cubic spline. Phase‐corrected Fourier transforms (FT) of the Extended X‐ray Absorption Fine Structure (EXAFS) data were extracted using the EXCURVE code and a k^2^ weighing scheme.^[^ ^96^ ^]^

### XPS

For the XPS measurements, the samples were treated as follows: a freshly prepared pellet of the Ti_3_Al_(1‐x)_Sn_x_C_2_ (x = 0.7) pristine MAX phase was broken into pieces and immersed in HCl 6M for 2 h, to remove oxidized metal impurities. After thorough rinsing with distilled water, several pieces of the pellets were attached via a conductive double‐sided carbon tape to the XPS sample holder to directly expose only the transversal section to the X‐ray beam. Care was taken in avoiding impinging of the X‐ray beam onto the external surface of the pellet to rule out signals of graphite punch residuals from the plasma sintering process. The powder of TiO_2_/Ti_(1‐y)_Al_z_Sn_y_O_2_ resulting from oxidation of the MAX phase at 700 °C (SnHigh_Ox700) was spread onto the conductive double‐sided carbon tape of the XPS sample holder. XPS measurements were carried out using a modified Omicron NanoTechnology MXPS system equipped with a monochromatic Al Kα (hν = 1486.7 eV) X‐ray source (Omicron XM‐1000), operating the anode at 14 kV and 16 mA. For each sample, the following binding energy (BE) regions were recorded: survey, Ti 2p, Sn 3d, Al 2p, C 1s, O 1s. The survey and Al 2p regions were acquired with an analyzer pass energy of 50 eV, while the other ones with a pass energy of 20 eV. In all measurements, the photoelectrons were collected at a take‐off angle of 21° relative to the normal of the sample surface.

The experimental spectra were theoretically reconstructed by fitting the secondary electrons background to a Shirley or linear function, and the elastic peaks to Voigt functions with a 30% Lorentzian weight. All peaks were symmetric, except those related to the elements of the nominal pristine MAX phase formula (Ti_3_Al_(1‐x)_Sn_x_C_2_, x = 0.7), reasonably displaying metallic behavior. In this case, a slight tailing function toward high BE was added as a factor to the Voigt function of these components.^[^ ^56^ ^]^ The relative amount of the different elements and chemical species was determined through area ratios with an uncertainty of ± 10%. Peak areas were normalized to the corresponding orbital ionization cross‐section according to Scofield calculations^[^ ^97^ ^]^ and for the square root dependence of the photoelectron kinetic energy.

### CHNS

The quantities of carbon present in the investigated samples were obtained by CHNS analysis, with an Elementar‐vario MACRO cube analyzer.

### SEM

The morphology of the SnHigh_Ox700 particles was characterized by the SEM Zeiss Gemini electron microscope at the University of Milano‐Bicocca.

### TEM

The whole TEM characterization was carried out at Ulsan National Institute of Science and Technology (UNIST). For the standard and ex situ HRTEM measurements, a JEOL JEM 2100F microscope was employed at an operating voltage of 200 kV. The three samples of SnHigh_Ox700 measured ex situ were prepared as follows: after cycling in coin cells versus Li (same preparation and characteristics of the electrochemical measurements), the materials were lithiated down to 0.7 V, 0.01 V, and finally delithiated back to 3 V. The three cells were opened in an argon‐filled glovebox, and the active materials were recovered by treating the electrodes with N‐methyl‐2‐pyrrolidone (NMP) to dissolve the binder. The recovered powders were then washed again with NMP and centrifuged three times to eliminate the binder and the carbon additive. The recovered active materials were then drop‐casted on TEM grids, and a Cryo‐TEM sample holder (Double tilt LN2 Atmos Defend Holder, Mel‐Build) was employed to transfer the samples in a controlled atmosphere and for the imaging measurements without beam damage. The information on the d‐spacing of the crystal structures was obtained by radially averaging the FFT of the HRTEM images, which provides a graph of the intensity as a function of the d‐spacing. The intensities were compared with the XRD reference cards from the ICDD PDF‐4+ 2023 database. For the in situ TEM measurements, the JEOL JEM 2100 Plus microscope was used at 200 kV with the two‐electrode Dual‐Probe STM‐TEM in situ holder from Nanofactory Instruments. SnHigh_Ox700 particles attached to a Cu tip formed the working electrode, while the counter/reference electrode was composed of a tungsten tip in contact with lithium metal. The naturally occurring Li_2_O thin layer acted as a solid electrolyte. Lithiation was performed by applying a 1.5 V bias between the two electrodes.

### Mössbauer spectroscopy

^119^Sn Mössbauer spectra were acquired in constant acceleration mode on powders (experiments at different degrees of oxidation) and electrodes (ex situ experiments upon cycling). The Ca^119m^SnO_3_ source and the absorber were kept at room temperature in all acquisitions. A LiF scintillation detector was employed for the detection of the γ‐rays. The velocity scale was calibrated with a high‐purity α‐Fe foil at room temperature using a ^57^Co:Rh source. The isomer shifts were referred to the CaSnO_3_ source. For the ex situ Mössbauer spectroscopy measurements, free‐standing electrodes were prepared by mixing active material with vapor‐grown carbon fiber (VGCF) and PTFE with a weight ratio of 75:20:5, respectively. Two‐electrode cells (ECC‐std cell of EL‐CELL®) were assembled with the so‐obtained electrodes, using metallic lithium as counter‐electrode, LP30 as electrolyte, and Whatman glass fiber as separator. The cells were cycled at 10 mA g^−1^ up to the different potentials of interest (0.7 V in lithiation, 0.01 V at the end of lithiation, 3 V at the end of delithiation). The spectra were analyzed by a standard least‐squares fitting procedure using Lorentzian quadrupole doublets. In this way, hyperfine parameters such as isomer shift (δ), quadrupole splitting (Δ), full linewidth at half‐maximum (Γ), and relative resonance areas of the different spectral components were determined. The fitted parameters are reported in Table S5 (Supporting Information).

### Electrode Fabrication

The electrodes were fabricated by mixing MAX phase powder with Super P carbon and a 4 wt% solution of poly(acrylic acid) (PAA) in NMP. A mixture of active material, carbon, and binder with a weight ratio of 92:5:3, was blended using a THINKY MIXER ARE‐310 at 1300 rpm for 5 min. NMP was added up to a solvent‐to‐slurry mass ratio of 70% to achieve the ideal slurry density. The slurry was then tape‐casted onto copper foils (MTI, thickness 9 µm) with a doctor blade to obtain a wet thickness of 10 mils (254 µm). The films were dried in vacuo at 120 °C for 12 h and subsequently calendared. The active material loading was ≈6–7 mg cm^−2^.

For full cell assembly, a commercial LiNi_0.8_Mn_0.1_Co_0.1_O_2_ (NMC 811, MTI Corp.) cathode prepared similarly, by using a 5 wt% solution of polyvinylidene difluoride (PVDF) in NMP, was used. The suspension was tape‐casted on aluminum foil (MTI, thickness 15 µm) with a doctor blade to achieve a wet thickness of 15 mils (381 µm). The electrode was dried under vacuum at 120 °C for 12 h and then calendared. The active material loading was ≈9–10 mg cm^−2^.

### Electrochemical Measurements

The electrochemical performance of the materials was evaluated in a half‐cell configuration. CR2032 coin cells (MTI) were assembled in an argon glovebox. The electrodes were cut into 16 mm diameter disks. Commercial LP30 (1 M LiPF6 in ethylene carbonate (EC) and dimethyl carbonate (DMC) in a 1:1 volume ratio) from MERCK was used as the electrolyte, and a 16 mm diameter Whatman glass fiber as the separator. The cells were cycled using a multichannel Arbin Lbt21084 at different gravimetric currents with the following standard protocol: 5 initial cycles at 15 mA g^−1^, followed by 10 cycles at each current (50, 100, 250, 500, 1000, 2000, and back to 50 mA g^−1^). Long‐term stability tests were carried out, repeating the following protocol three times: 10 cycles at 50 mA g^−1^ plus 300 cycles at 100 mA g^−1^.

For the full cell tests, a three‐electrode Hohsen cell was used. The cell was assembled with SnHigh_Ox700 as the negative electrode, NMC 811 as the positive electrode, Li as the reference electrode, LP30 as the electrolyte, and Whatman as the separator. The electrodes were cut into 10 mm diameter disks. The N/P ratio can be calculated to be ≈0.7, assuming specific capacitance values of 330 mAh g^−1^ for N (from experimental data) and 200 mAh g^−1^ for P (theoretical data considering LiNi_0.8_Mn_0.1_Co_0.1_O_2_). The negative electrode was prelithiated for 2 cycles at 15 mA g^−1^ to avoid irreversible capacity loss in the first cycle, including SEI formation. The full cell was cycled for 10 cycles at 20 mA g^−1^ (C/10 relative to the positive electrode material) and then for 70 cycles at 40 mA g^−1^ using the multichannel Bio‐Logic VSP‐300.

## Conflict of Interest

The authors declare no conflict of interest.

## Supporting information



Supporting Information

Supplemental Video 1

Supplemental Video 2

## Data Availability

The data that support the findings of this study are available from the corresponding author upon reasonable request.
