# Supplementary material for: Tailoring Oxide/MAX Phase Nanocomposites via Low‐Temperature Oxidation for Lithium‐Ion Battery Anodes: Peeking Behind the Electrochemical Mechanism via In Situ Investigations
Source: Adv Sci (Weinh). 2025 Sep 24;12(45):e12947. doi: 10.1002/advs.202512947 (PMC12677627; doi:10.1002/advs.202512947)
Supplement: Supplementary file 1 — Supporting Information [file ADVS-12-e12947-s003.pdf]

Supporting Information

Tailoring oxide/MAX Phase nanocomposites via low-temperature oxidation for lithium-ion battery anodes: peeking behind the electrochemical mechanism via *in-situ* investigations

*Irene Ostroman,† Nicholas Vallana,† Antonio Gentile, Stefano Marchionna, Omar Perego, Chiara Ferrara, Andrew Fitch, Martina Fracchia, Nicolò Pianta, Andrea Giacomo Marrani, Taewon Kim, Changhyun Park, Chanhee Lee, Hyun-Wook Lee, Lorenzo Stievano, Riccardo Ruffo**

1. Oxidation reaction as function of the oxidation degree and as function of the Sn content

$$Ti_3Al_{1-y}Sn_yC_2 + \left(\frac{y}{4}+6\right) x\, O_2 \rightarrow (1-x)Ti_3Al_{1-y}Sn_yC_2 + 3x\, TiO_2 + \frac{1-y}{2} xAl_2O_3 + xy\, SnO_2 + 2x\, CO_2$$

Eq. S1

With the ultimate goal of controlling the phase and chemical composition of the MAX/oxides nanocomposite, two main parameters have been identified as relevant: i) the advancement degree of the oxidation (x in Eq. S1); ii) the starting Al/Sn ratio (y in Eq. S1).
The former controls the ratio among the MAX phase and the oxides formed while the latter controls the TiO_2 / SnO_2 ratio. The variation of these two parameters is represented in Figure S1.

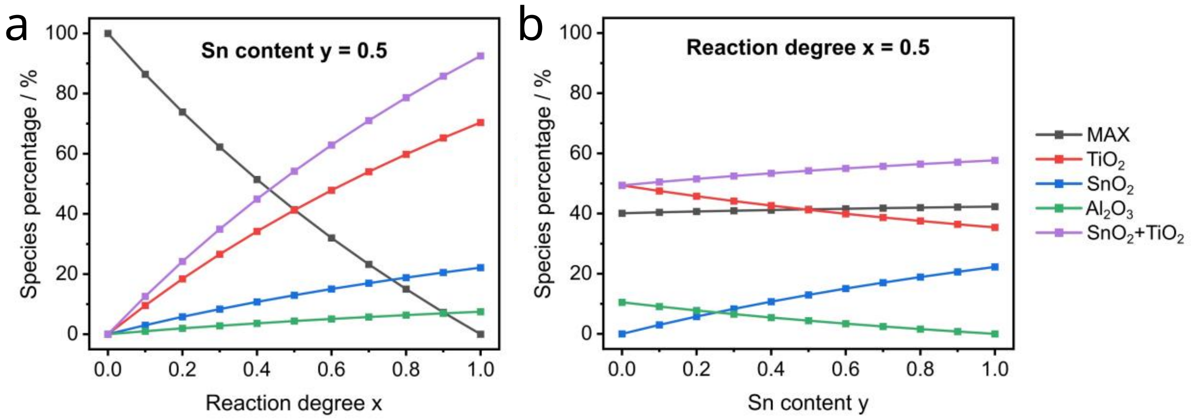

Figure S1. Evolution of the wt% of the MAX phase, TiO_2, SnO_2, Al_2O_3, and the total fraction of active oxides according to Equation S1. (a) Variation as a function of the reaction degree x (extent of oxidation) with fixed Sn content y = 0.5 (corresponding to initial MAX composition $Ti_3Al_{0.5}Sn_{0.5}C_2$). (b) Variation as a function of Sn content y in the initial MAX composition, with fixed oxidation advancement x = 0.5.

As obvious, as the oxidation reaction proceeds (variation of x), the MAX phase is consumed in favor of the production of oxides, as shown in Figure S1a. Nevertheless, this trend is relevant as, for the final application, it is essential to optimize the relative amounts of conductive MAX phase, electrochemically active TiO_2/SnO_2, and inert Al_2O_3. In this regard, the range $0.4 < x < 0.6$ appears to be the more favorable. Experimentally the values explored are within this range, as reported by crosses in Figure S1a.
The optimization of the Al/Sn ratio is particularly important as the $(Ti/Sn)O_2$ system is the active species, whereas Al_2O_3 is electrochemically inert. Increasing the Sn content therefore leads to some beneficial effects: i) lowering the thermal resistance of the systems, ii) lowering the final Al_2O_3 content and iii) increasing the capacity of the final composite. Formally, the Sn-richest $Ti_{1-z}Sn_zO_2$ composition should correspond to z = 0.25, given the 3:1 ratio in the Ti_3SnC_2 end member compound.

The composition of the samples obtained by annealing at increasing temperatures can be estimated from the mass values and the mass growth during the oxidation processes. Such values, reported in **Table S1** and illustrated in **Figure 2a**, were rationalized based on two main assumptions: i) the samples are constituted uniquely of the 312 MAX phase, neglecting the 211 fraction; ii) the oxidation evolves according to Equation 1. This way, it is possible to estimate the value of x in Equation 1, and consequently the relative amounts of oxides formed during the oxidation process. Under these assumptions, sample SnHigh_Ox600 is composed of 42 wt% residual MAX phase and 55 % Ti/Sn oxides. The amount of Ti/Sn oxides increase to 77 wt% in SnHigh_Ox700, and to 92 % in SnHigh_Ox850. A stable Ti/Sn ratio can be derived from the composition initial MAX phase, roughly corresponding to an average $Ti_{0.8}Sn_{0.2}O_2$ stoichiometry.

Table S1. Weight percent of MAX phase and oxide species determined by analyzing the relative amounts of the three samples, and assuming the validity of Equation 1, together with the mass percentage of Carbon, as evaluated via CHNS analysis.

	**Carbon wt% (CHNS)**	**MAX / wt%**	**TiO_2 / wt%**	**SnO_2 / wt%**	**Al_2O_3 / wt%**	**Tot/$(Ti/Sn)O_2$ / wt%**
**SnHigh_Ox600**	3	37	41	16	3	57
**SnHigh_Ox700**	2	17	56	21	4	75
**SnHigh_Ox850**	0	4	66	25	5	91

2. X-ray diffraction: data and analysis

Table S2. Results from Rietveld refinements of the XRD data obtained at room temperature for the SnHigh and for the SnHigh_Ox700 samples. The analysis of the SnHigh_Ox700 sample has been based on the presence of 312, 211, TiC phases as they are present in the pristine SnHigh composition; in addition two $(Ti/Sn)O_2$ phases with the same crystal structure ($P4_2/mnm$) and different cell parameters have been introduced to account for all the reflections in the pattern and have been labelled "$(Ti/Sn)O_2$_highSn and "$(Ti/Sn)O_2$_highTi as the cell parameters are in between the range defined by the SnO_2 cassiterite structure and TiO_2 rutile

structure. Al_2O_3 at this stage should be formed as well but it is not detected in the pattern; this suggests it is not in crystalline form and/or it is nanometric.

	SnHigh	SnHigh_Ox700
**Phase quantification / wt%**		
**312**	92.58(3)	22.6(8)
**211**	6.71(6)	5.6(4)
**TiC**	0.71(1)	0.3(2)
**(Ti/Sn)O_2 – highSn**	-	27.5(5)
**(Ti/Sn)O_2 – highTi**	-	44(1)
**Rwp; Chi2**	15.3, 6.48	7.15, 10.32
**312 cell parameters**		
**a / Å**	3.10904(1)	3.1072(2)
**c / Å**	18.61776(5)	18.632(3)
**V / Å^3**	155.851(6)	155.79(3)
**Sn/Al / %**	0.57:0.43	-
**211 cell parameters**		
**a / Å**	3.11716(6)	3.1229(6)
**c / Å**	13.71597(3)	13.699(3)
**V / Å^3**	115.418(4)	115.70(4)
**Sn/Al / %**	0.62:0.38	-

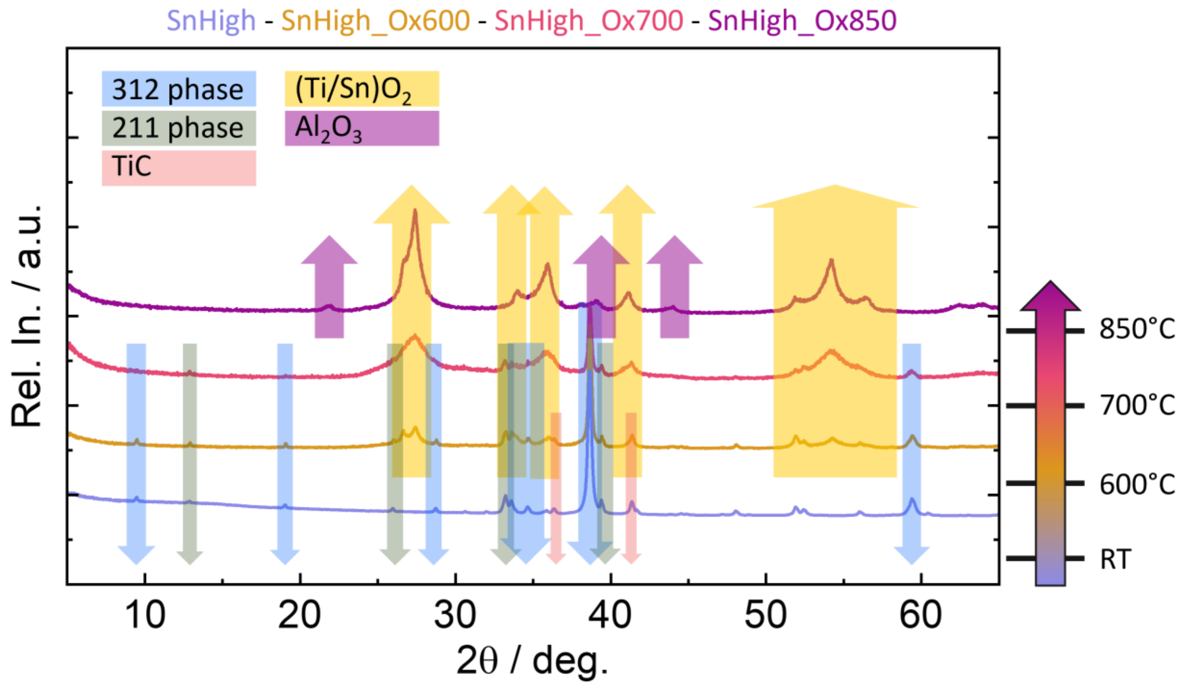

Figure S2 – XRD patterns for the SnHigh, SnHigh_Ox600, SnHigh_Ox700, SnHigh_850 with highlight on the phase evolution with the MAX phases decreasing and oxide increasing with the preparation temperature.

Table S3 – Phase quantification for the SnHigh, SnHigh_Ox600, SnHigh_Ox700, SnHigh_850 derived from Rietveld refinement analysis.

	**SnHigh**	**SnHigh_Ox600**	**SnHigh_Ox700**	**SnHigh_Ox850**
**312**	92.58(3)	58.26(5)	22.6(8)	-
**211**	6.71(6)	16.51(4)	5.6/4)	-
**TiC**	0.71(1)	0.16(5)	0.3(2)	-
**$(Ti/Sn)O_2$**	-	25(1)	71(1)	93(3)
**Al_2O_3**	-	-	-	7(1)

3. Spectroscopic analyses

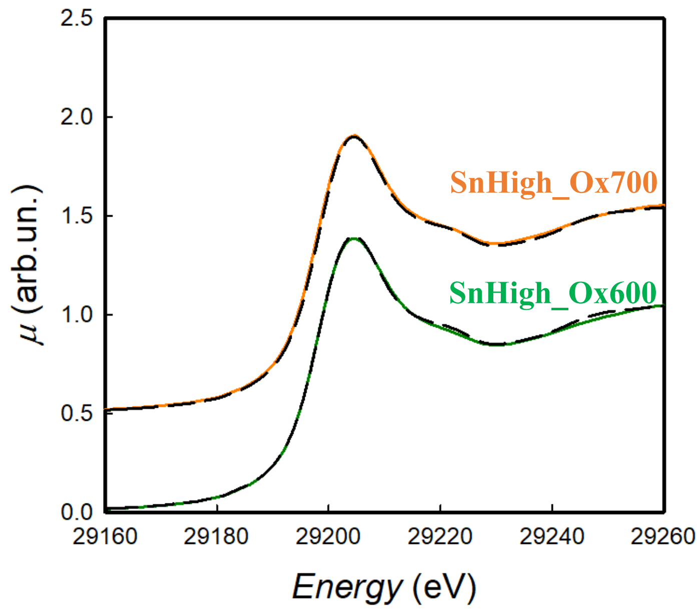

Figure S3. XANES spectra at the Sn K-edge of the SnHigh_Ox600 and SnHigh_Ox700 samples and linear combination fittings (dashed lines), obtained starting from the spectra of SnO_2 and the pristine MAX phase.

Table S4. BE position (eV) and chemical species assignments of the relevant ionization features in the XPS spectra of the two MAX phase samples. Area % values are also reported.

**Ti 2p$_{3/2}$**				
	**Ti(+1)** $Ti_3Al_{(1-x)}Sn_xC_2$ **/ area %**	**Ti(+2)** *TiO* **/ area %**	**Ti(+3)** Ti_2O_3 **/ area %**	**Ti(+4)** TiO_2 **/ area %**
**MAX pristine**	454.6 / 39.7	455.44 / 28.6	456.85 / 20.1	458.87 / 11.6
**MAX ox***	-	-	-	458.70
**Sn 3d$_{5/2}$**				
	**Sn(0)** $Ti_3Al_{(1-x)}Sn_xC_2$ **/ area %**	**Sn(+2+y)** SnO_{2-x} **/ area %**	**Sn(+4)** SnO_2 **/ area %**	***satellite*** **/ area %**
**MAX pristine**	484.92 / 63.1	-	487.45 / 36.9	-
**MAX ox**	-	486.54 / 60.4	487.74 / 27.6	492.15 / 12.0
**C 1s**				
	**carbide** $Ti_3Al_{(1-x)}Sn_xC_2$ **/ area %**	**CH$_x$, graphite / area %**	**C-OH / area %**	**COOH / area %**
**MAX pristine**	281.82 / 22.0	285.00 / 58.2	286.56 / 13.0	289.08 / 6.8
**MAX ox**	-	285.00 / 64.0	286.54 / 20.1	289.00 / 15.9
**Al 2p$_{3/2}$**				
	**Al(0)** $Ti_3Al_{(1-x)}Sn_xC_2$		**Al(+3)** Al_2O_3	
**MAX pristine**	72.45		-	
**MAX ox**	-		74.20	
*Satellites at 460.22, 463.00 and 471.65 eV				

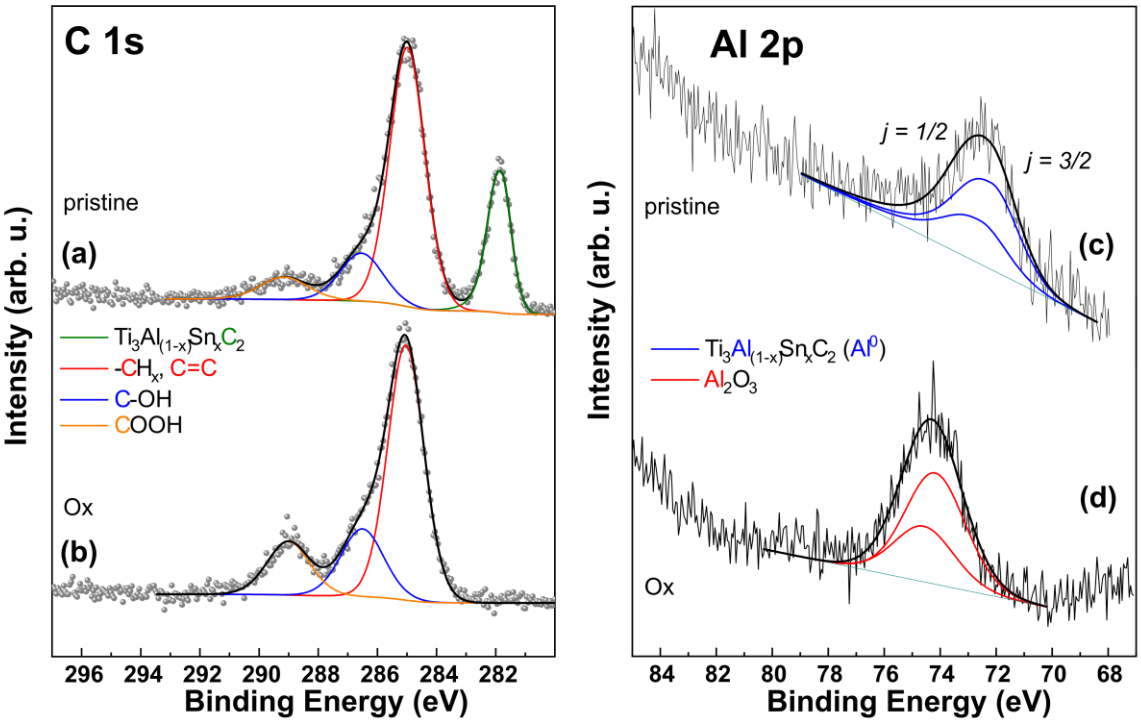

Figure S4. XPS spectra of the MAX pristine and oxidized samples in the C 1s (a,b) and Al 2p (c,d) photoionization regions.

Table S5. Room temperature hyperfine Mössbauer parameters of the MAX phase in the pristine state and after thermal treatment at increasing temperatures. The spectra were fitted at the same time, with common hyperfine parameters for the two components representing the MAX phase. The isomer shift is given relative to $BaSnO_3$ at 300 K.

Sample	δ (mm/s)	Δ (mm/s)	Γ (mm/s)	Relative area (%)	Tin species
**SnHigh**	1.92(1)	1.97(1)	0.98(4)	69(6)	MAX phase 1
	2.25(4)	1.91(4)	1.62(5)	31(6)	MAX phase 2
**SnHigh_Ox600**	1.92(1)	1.97(1)	0.98(4)	47(5)	MAX phase 1
	2.25(4)	1.91(4)	1.62(5)	15(5)	MAX phase 2
	0.34(9)	-0.05(2)	1.02(9)	38(1)	Sn(IV)
**SnHigh_Ox700**	1.92(1)	1.97(1)	0.98(4)	42(3)	MAX phase 1
	2.25(4)	1.91(4)	1.62(5)	11(3)	MAX phase 2
	0.52(1)	-0.06(1)	0.96(2)	47(1)	Sn(IV)
**SnHigh_Ox850**	0.60(1)	0.01(1)	1.23(2)	100	SnO_2

4. Long cycling analysis

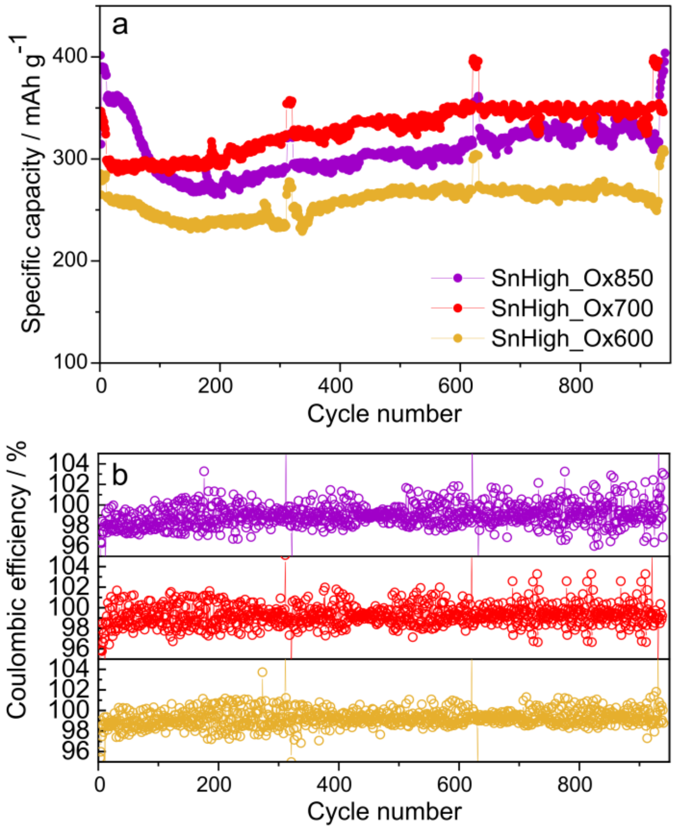

Figure S5. Long cycling measurements of SnHigh_Ox600, SnHigh_Ox700, and SnHigh_Ox850, specific capacity in de-lithiation (a) and Coulombic efficiency (b). The protocol applied consists of 10 cycles at 50 mA g^{-1} followed by 300 cycles at 100 mA g^{-1}, repeated 3 times.

5. TEM analyses on pristine SnHigh_700Ox

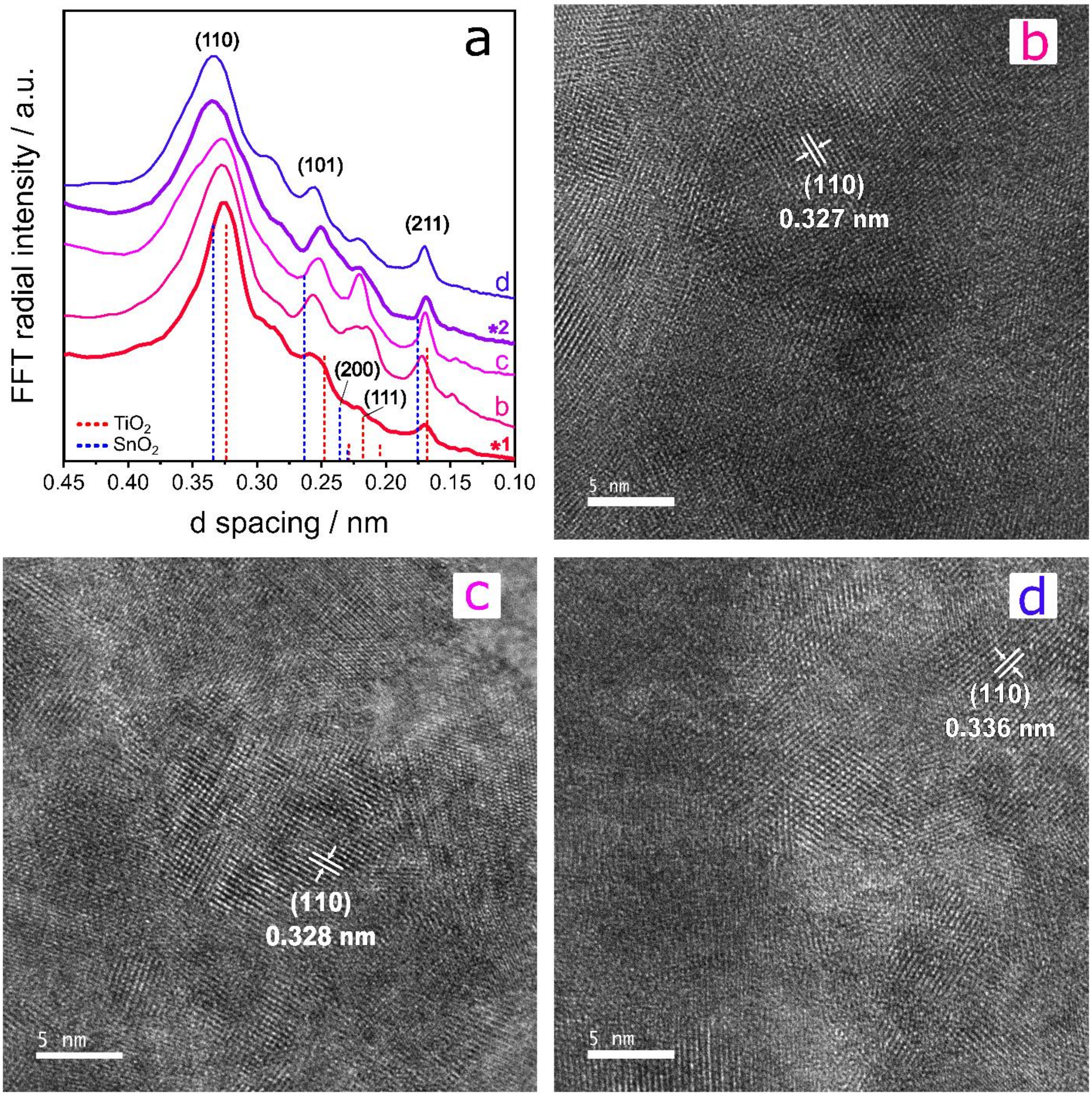

Figure S6. TEM analysis of pristine SnHigh_Ox700 sample. FFT radial intensity as a function of the d-spacing (a) obtained from HRTEM images reported in this image (blue reference line: SnO_2 (PDF card 01-087-9075); red reference line: rutile TiO_2 (PDF card 00-034-0180)). The intensity lines in panel (a) are referred to the corresponding panels in this image, except for the two ones marked with asterisks (*[1] and *[2]), which are referred respectively to Figure 3f and 3h from the main text.

6. Operando analyses and results

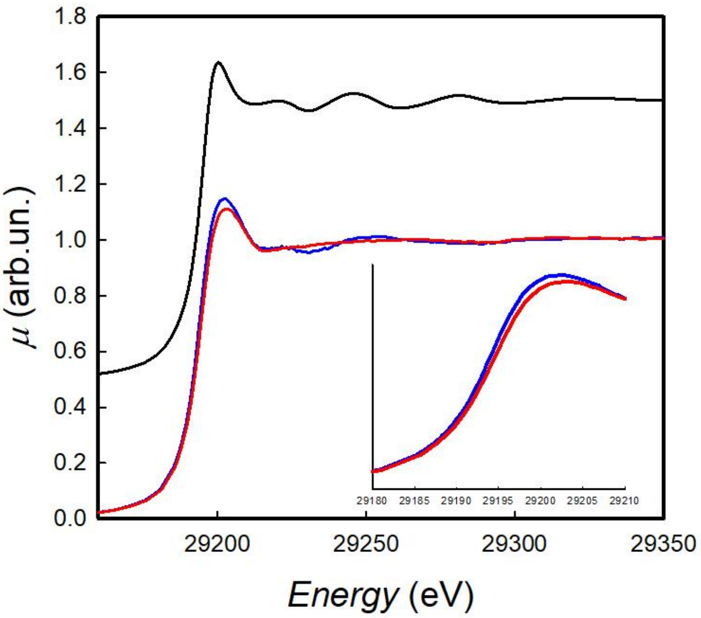

Figure S7. XAS spectra at the Sn K-edge of the SnHigh_Ox700 sample recorded during the lithiation process at 0.4 V (blue line) and 0.01 V (red line). The inset shows a zoom in the XANES region, allowing to better highlight the shift at higher energies of the spectrum at 0.01 V. The spectrum of the tin foil is also shown for comparison.

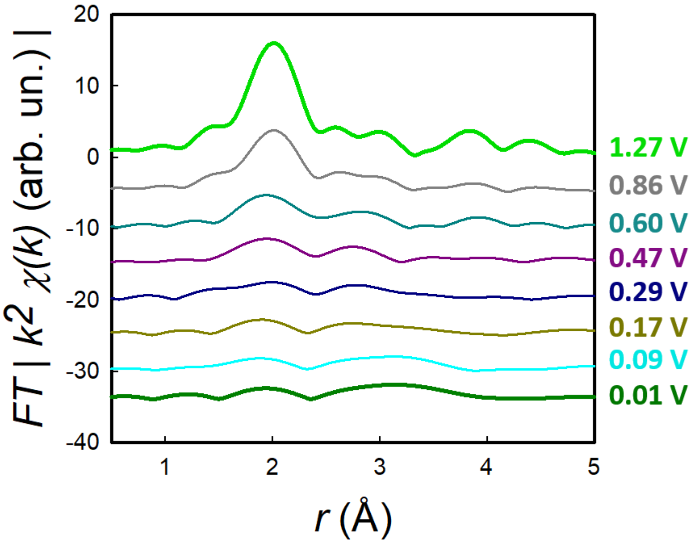

Figure S8. Phase-corrected Fourier Transforms of the EXAFS spectra at selected potential values during the lithiation process.

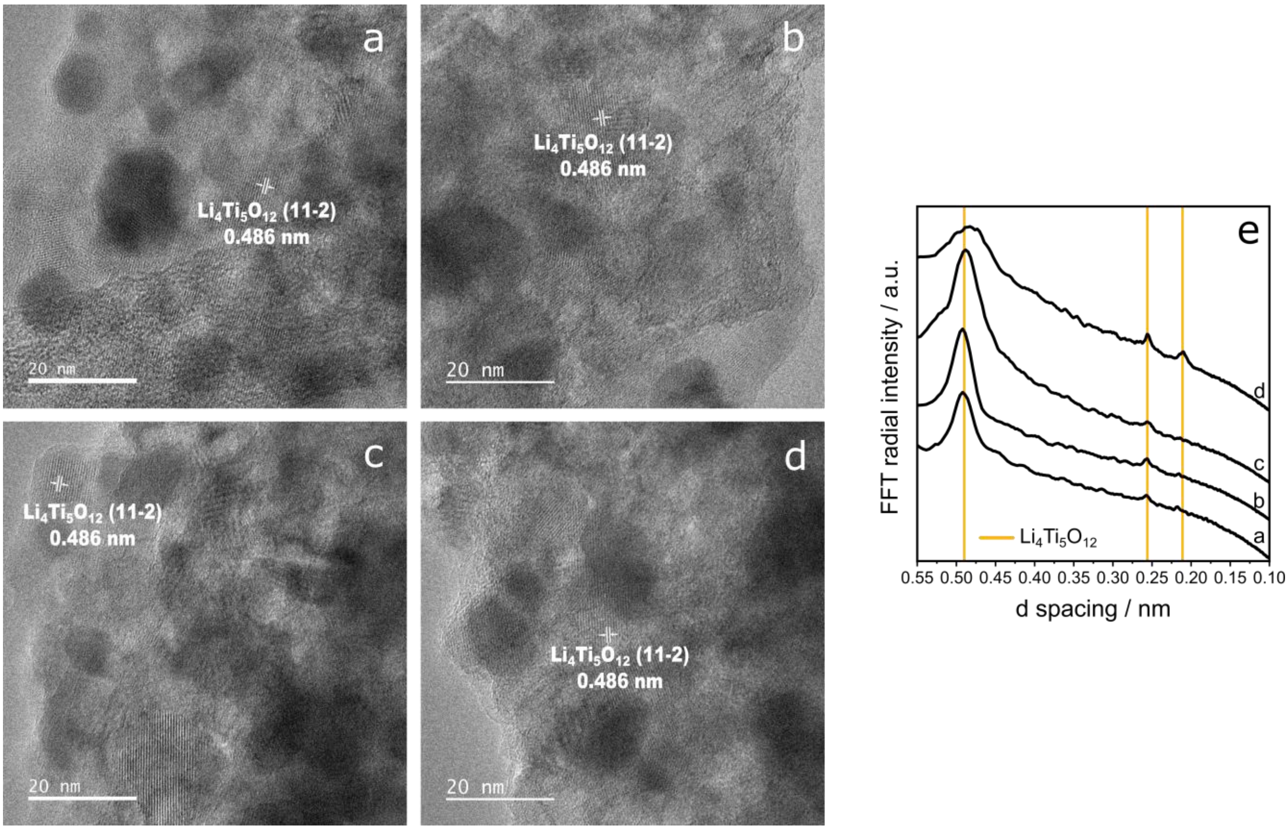

Figure S9. HRTEM analysis of SnHigh_Ox700 sample cycled and stopped at 0.7 V during first lithiation (a-d) and corresponding FFT radial intensity as a function of the d-spacing (e). Yellow line: $Li_4Ti_5O_{12}$ (PDF card 49-0207).

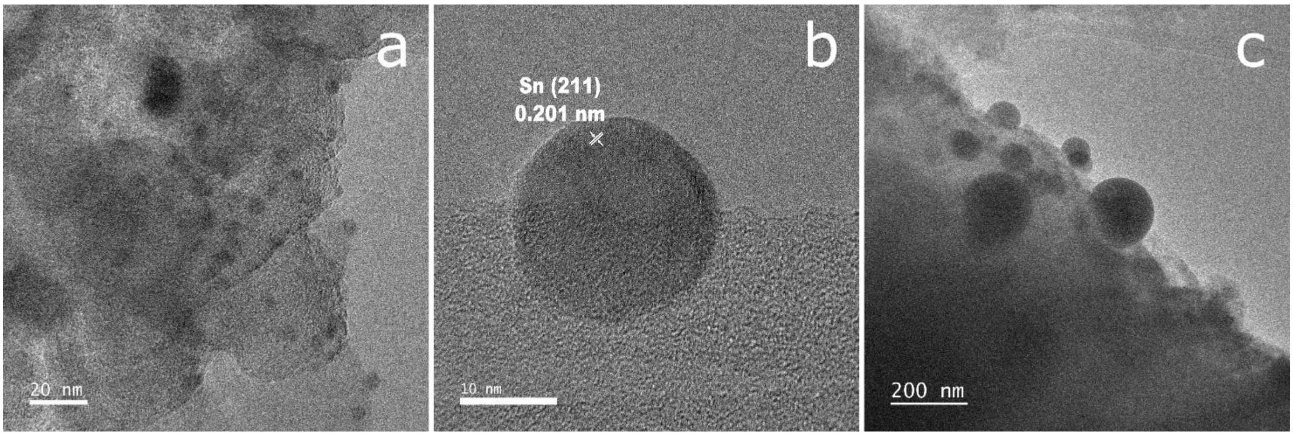

Figure S10. TEM images of the sample cycled and stopped at 0.7 V during lithiation.

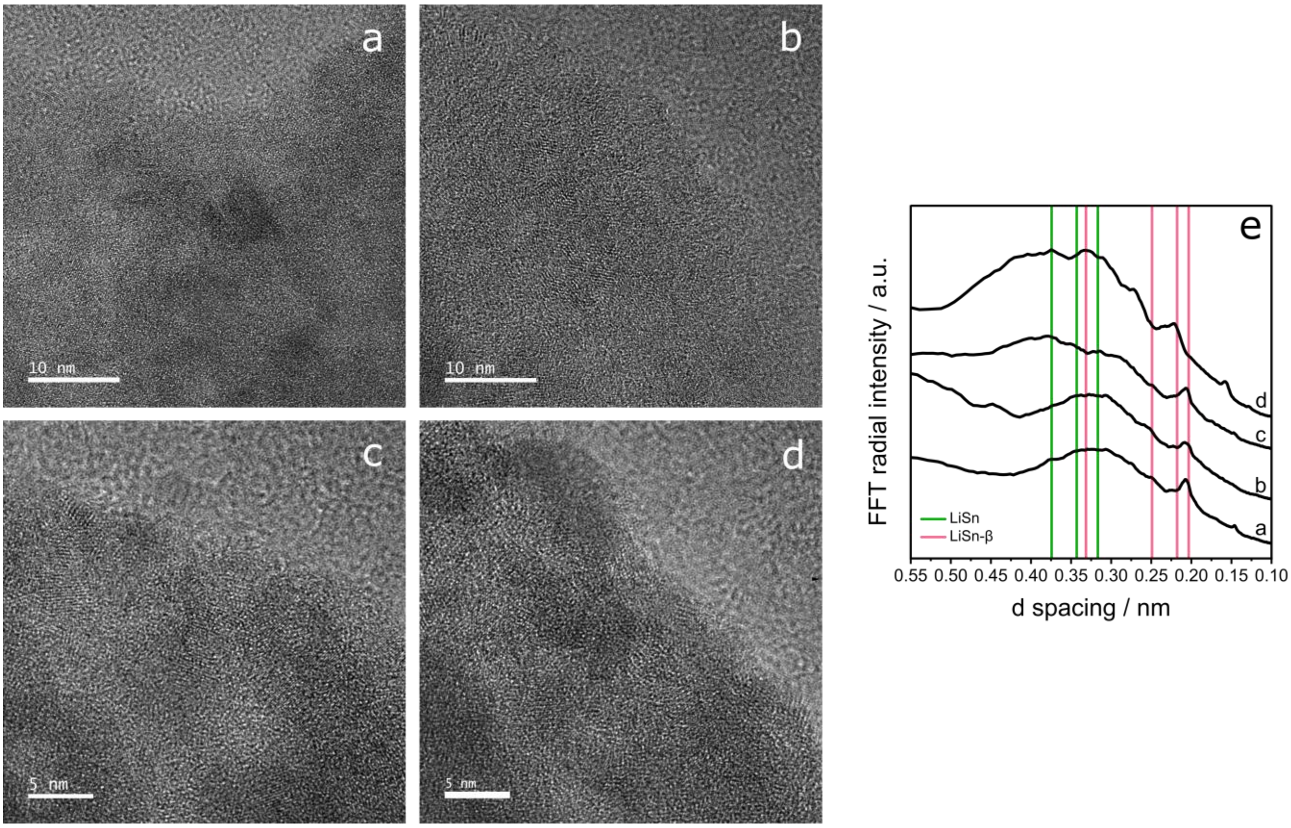

Figure S11. HRTEM analysis of SnHigh_Ox700 sample cycled and stopped at the end of the first lithiation at 0.01 V (a-d) and corresponding FFT radial intensity as a function of the d-spacing (e). Green line: LiSn (PDF card 98-010-4782). Pink line: LiSn-β (PDF card 98-010-7516).

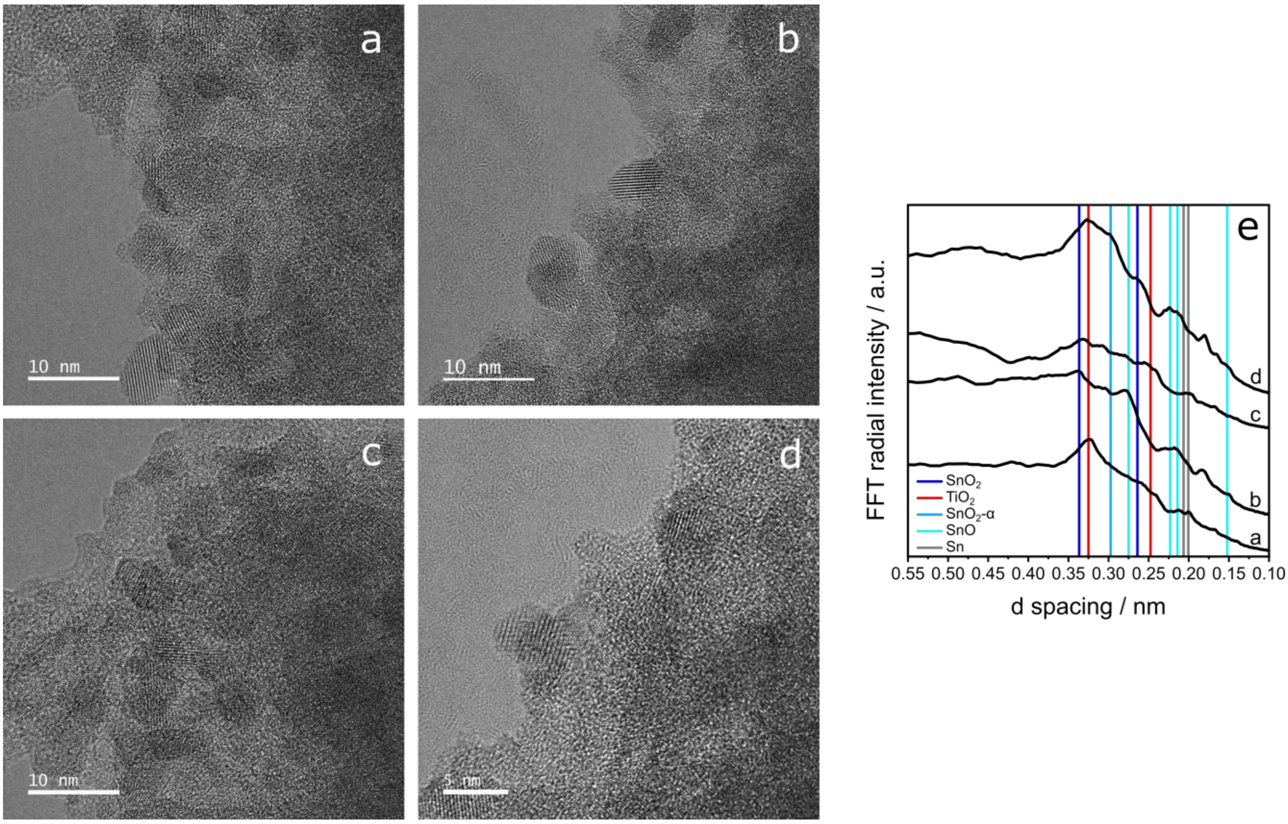

Figure S12. HRTEM analysis of SnHigh_Ox700 sample cycled and stopped at the end of the first delithiation at 3 V (a-d) and corresponding FFT radial intensity as a function of the d-spacing (e). Blue line: SnO_2 (PDF card 01-087-9075). Red line: rutile TiO_2 (PDF card 00-034-0180). Light blue line: SnO_2-α (PDF card 98-015-7450). Cyano line: SnO (PDF card 98-002-0624). Gray line: metallic Sn (PDF card 00-001-0926).

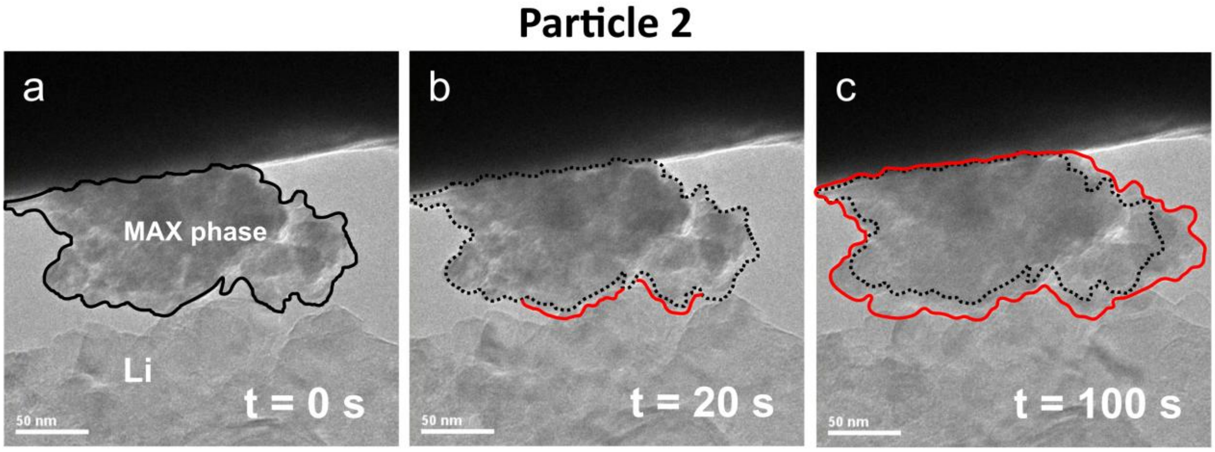

Figure S13. A sequence of images taken with operando TEM of a SnHigh_Ox700 grain (Particle 2) upon lithiation: no contact and no biasing, particle area = 1.6×10^4 nm^2 (a); 20 s after applying bias (b); 100 s after starting bias, particle area = 2.1×10^4 nm^2 (c).

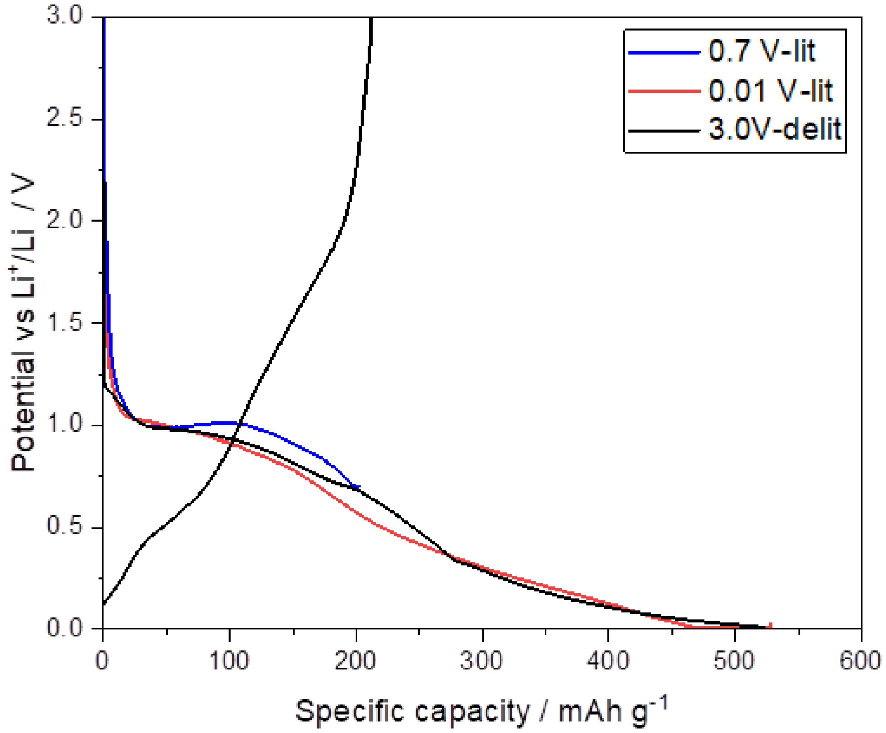

Figure S14. Cycling profile of the electrodes prepared for the ex situ Mössbauer spectroscopy experiments.

7. Comparison between SnHigh_Ox700 and other similar anodes.

Table S6. Comparison between the electrochemical performance of several anode materials based on Tin and/or Titanium found in literature (together with the compound proposed in this work).

	Sn quantity / wt%	SnO_2 quantity / wt%	TiO_2 quantity / wt%	Anodic capacity 1st cycle / mAh g^{-1}	Coulombic efficiency 1st cycle / %	Current / mA g^{-1}	Stable anodic capacity / mAh g^{-1}	Coulombic efficiency / %	Cycle number	Capacity retention / %	Reference
TiO_2/graphene nanoribbons	-	-	40.7 (anatase), 59.3 (TiO_2(B))	390	79.5	335	131	99.2	1400	95.1	[1]
2D C@0D nanosheets TiO2@SnO2 nanoparticles	-	20	36	707	51.5	200	758	>90	400	100	[2]
						1000	474	NR	650	100	
TiO_2/C	-	-	80.1	330.9	60.1	84	630.3	99.71	400	100	[3]
						336	271.6	99.4	500	100	
Mo/Sn–Mo/Mo	NR	-	-	870	84	200	605	98	200	86	[4]
$Sn_{55}Zn_{45}$	69.0	-	-	1200	NR	50	600	NR	100	60	[5]
Sn/Fe/C	49.4	-	-	894.6	63.4	500	400	>95	1100	90.9	[6]
Sn/Ti/C nanofibers	NR	-	NR	475	73	50	557	>95	100	98.7	[7]

SnCo/CNF	53.25	-	-	1602	62	50	788	NR	50	NR	[8]
Sn/lignite	83	-	-	798	64	1000	573	NR	800	100	[9]
Flakes Sn/SnO_2/C	NR	NR	-	1200	70	1000	840	>90	1000	NR	[10]
Sn/SnO2/C	33.2	52.3	-	752.2	79.9	100	683.3	99.74	200	90.8	[11]
						1000	444.5	99.63	1000	NR	
SnO_2-PNT nanotubes	-	NR	-	1500	55.91	1000	789.6	>99	1000	NR	[12]
SnO_2 nanospheres	-	100	-	1013.8	66.2	500	1009.9	>95	300	NR	[13]
Hollow SnO_2@C nanoparticles	-	NR	-	831	57.3	200	769	>90	350	98.6	[14]
3D porous SnO_2/C	-	35	-	571.4	45.93	250	513	>90	250	92.5	[15]
						500	284.56	>90	400	NR	
SnO_2/C	-	55.96	-	966	61.85	1000	798	98.68	100	NR	[16]
SnO_2/C nanorods	-	88.36	-	1138	65.23	1000	1007	98.34	100	NR	[16]
SnO_2/C	-	28.88	-	1050	68.64	2000	600	>90	1000	NR	[17]
SnO_2nanocrystals/C	-	NR	-	1194	72.66	200	1011.2	>90	160	NR	[17]
						1000	666.4	NR	400	NR	
W-SnO2/graphite nanosheets	-	83	-	1070	76.2	200	1148.8	>98	500	100	[18]
						1000	1229.5	>95	800	100	
SnO/graphene 2D-heterostructures	-	66 (SnO)	-	954.6	67.63	50	633.9	NR	100	NR	[19]
						1000	517.2	>90	700	NR	
SnO_x/ZnO/C nanofibers	-	28	-	827.9	73.2	500	588.7	>90	100	NR	[20]
						2000	300	>90	2000	NR	
SnO_2/MXene	-	50	-	695±50	56.5±3.5	100	525±25	98.8±0.3	700	100	[21]
SnO2 quantum dots/MXene	-	NR	-	637	60.9	50	506	NR	160	98	[22]
						1000	403	NR	160	98	
SnO2 nanoparticles/MXene	-	NR	-	961	60	100	904.1	>95	1000	100	[23]
						1000	620.8	NR	500	100	
Sphere-like SnO_2/TiO_2	-	85	15	900	62	500	483	NR	40	NR	[24]
SnO_2/TiO_2	-	NR	NR	600	55	453	307	>90	300	NR	[25]
Sn_3O_4/TiO_2 nanobelts	-	85.7 (Sn_3O_4)	14.3	1104.4	72.96	60	659	98.16	50	NR	[26]
SnO2 nanoparticles@ hollow TiO2 nanospheres/C	-	79	20	800	71	200	484	>90	300	69.4	[27]
SnO_2/TiO_2	5	15	35	427	51	100	270	98.5	150	98.5	[28]
Sn(SnO_2)/TiO_2(B)	20% Sn(SnO_2)	-	80	604.8	66	100	500	roughly 98	50	NR	[29]
						10000	188	NR	3000	NR	
						20000	117	NR	3000	NR	
SnO_2/TiO_2/C	-	65.67	21.89	1013	57.49	1000	908	99.82	100	NR	[16]
**SnO_2/TiO_2**	**-**	**21**	**56**	**347**	**54.1**	**100**	**330±18**	**99.2±1.9**	**930**	**100**	**This work**

References

[1] X. Li, Y. Liu, X. Zhang, C. Yao, R. Wang, C. Xu, J. Lei, *Electrochim Acta* **2019**, *298*, 14.
[2] Q. Tian, Y. Chen, F. Zhang, W. Zhang, Z. Sui, L. Yang, *Appl Surf Sci* **2020**, *511*, 145625.
[3] M. Han, Y. Mu, F. Yuan, X. Bai, J. Yu, *J Power Sources* **2020**, *465*, 228206.
[4] X. Lan, J. Cui, H. Yu, X. Y. Xiong, L. Tan, R. Hu, *J Power Sources* **2021**, *509*, 230391.
[5] X. Zhang, Y. Gong, C. Xu, P. Qu, G. Wang, *Ionics (Kiel)* **2022**, *28*, 230391.
[6] M. Chen, K. Yang, B. Dong, Q. Zhou, Y. Zhang, Y. Zhu, A. Iqbal, X. Liu, C. Yan, C. T. J. Low, X. Qian, *J Power Sources* **2023**, *553*, 232272.
[7] T. Li, Y. Chen, L. Wang, X. Xia, *Journal of Solid State Electrochemistry* **2020**, *24*, 781.
[8] X. Zhang, E. Qu, Q. Xiao, J. Zhang, G. Lei, Z. Li, J. Guan, *Ionics (Kiel)* **2019**, *25*, 5735.
[9] J. Zhu, Z. Zhang, X. Ding, J. pei Cao, G. Hu, *J Colloid Interface Sci* **2021**, *587*, 367.
[10] Q. Sun, X. Kong, W. Liu, B. Xu, P. Hu, Z. Gao, Y. Huang, *J Alloys Compd* **2020**, *831*, 154677.
[11] M. Han, Y. Mu, J. Yu, *Energy Technology* **2020**, *8*, 1901202.
[12] J. Man, K. Liu, Y. Du, J. Sun, *Mater Chem Phys* **2020**, *256*, 123669.
[13] J. Y. Cheong, J. H. Chang, C. Kim, J. Lee, Y. S. Shim, S. J. Yoo, J. M. Yuk, I. D. Kim, *ACS Appl Energy Mater* **2019**, *2*, 3.
[14] Q. Tian, Y. Chen, W. Zhang, J. Chen, L. Yang, *J Alloys Compd* **2020**, *820*, 153382.
[15] Z. Tian, J. Zhao, B. Li, Y. Feng, J. Song, C. Niu, L. Shao, W. Zhang, *Ionics (Kiel)* **2020**, *26*, 2773.
[16] A. Staffolani, L. Sbrascini, G. Carbonari, F. Maroni, L. Minnetti, L. Bottoni, F. Nobili, *Adv Mater Technol* **2025**, *10*, 2402058.
[17] S. Zhou, H. Zhou, Y. Zhang, K. Zhu, Y. Zhai, D. Wei, S. Zeng, *Nanomaterials* **2022**, *12*, 700.
[18] Y. Feng, K. Wu, J. Ke, X. Huang, C. Bai, H. Dong, D. Xiong, M. He, *Appl Surf Sci* **2020**, *533*, 147508.
[19] P. Jiang, J. Jing, Y. Wang, H. Li, X. He, Y. Chen, W. Liu, *J Alloys Compd* **2020**, *812*, 152114.
[20] L. Ao, C. Wu, Y. Xu, X. Wang, K. Jiang, L. Shang, Y. Li, J. Zhang, Z. Hu, J. Chu, *J Alloys Compd* **2020**, *819*, 153036.
[21] A. Gentile, S. Arnold, C. Ferrara, S. Marchionna, Y. Tang, J. Maibach, C. Kübel, V. Presser, R. Ruffo, *Adv Mater Interfaces* **2023**, *10*, 2202484.
[22] L. Wang, Y. He, D. Liu, L. Liu, H. Chen, Q. Hu, X. Liu, A. Zhou, *J Electrochem Soc* **2020**, *167*, 116522.
[23] C. Zhao, Z. Wei, J. Zhang, P. He, X. Huang, X. Duan, D. Jia, Y. Zhou, *J Alloys Compd* **2022**, *907*, 164428.
[24] R. Li, W. Xiao, C. Miao, R. Fang, Z. Wang, M. Zhang, *Ceram Int* **2019**, *45*, 13530.
[25] H. Yoo, G. Lee, J. Choi, *RSC Adv* **2019**, *9*, 12.
[26] X. Chen, Y. Huang, K. Zhang, X. Feng, M. Wang, *Electrochim Acta* **2018**, *259*, 131.
[27] S. Wang, X. Yu, J. Liu, P. Dong, Y. Zhang, C. Zhu, Z. Zhan, Y. Zhang, *J Alloys Compd* **2020**, *814*, 152342.
[28] S. Jolly, S. Husmann, V. Presser, M. Naguib, *Journal of the American Ceramic Society* **2023**, *106*, 3261.
[29] T. Autthawong, C. Yodbunork, N. Ratsameetammajak, O. Namsar, Y. Chimupala, T. Sarakonsri, *ACS Appl Energy Mater* **2022**, *5*, 11.
